# Polo-like kinase 1 is related with malignant characteristics and inhibits macrophages infiltration in glioma

**DOI:** 10.3389/fimmu.2022.1058036

**Published:** 2022-12-21

**Authors:** Lin Luo, Xiao-Yang Zhang, Ying-Wei Zhen, Gao-Chao Guo, Da-Zhao Peng, Cheng Wei, Dong-Ling Pei, Bin Yu, Yu-Chen Ji, Xian-Zhi Liu, Lei Han, Zhen-Yu Zhang

**Affiliations:** ^1^ Department of Neurosurgery, The First Affiliated Hospital of Zhengzhou University, Zhengzhou, Henan, China; ^2^ Academy of Medical Sciences, Zhengzhou University, Zhengzhou, Henan, China; ^3^ Tianjin Neurological Institute, Key Laboratory of Post-Neuro injury Neuro-repair and Regeneration in Central Nervous System, Ministry of Education and Tianjin City, Tianjin Medical University General Hospital, Tianjin, China; ^4^ Department of Neurosurgery, Zhengzhou University People’s Hospital, Henan Provincial People’s Hospital, Zhengzhou, Henan, China

**Keywords:** plk1, glioma, tumor immune microenvironment, immune infiltration, macrophages

## Abstract

**Background:**

Tumor immune microenvironment (TIM) plays a critical role in tumorigenesis and progression. Recently, therapies based on modulating TIM have made great breakthroughs in cancer treatment. Polo-like kinase 1 (PLK1) is a crucial regulatory factor of the cell cycle process and its dysregulations often cause various pathological processes including tumorigenesis. However, the detailed mechanisms surrounding the regulation of PLK1 on glioma immune microenvironment remain undefined.

**Methods:**

Public databases and online datasets were used to extract data of PLK1 expression, clinical features, genetic alterations, and biological functions. The EdU, flow cytometry, and macrophage infiltration assays as well as xenograft animal experiments were performed to determine the relationship between PLK1 and glioma immune microenvironment *in vivo* and *in vitro*.

**Results:**

PLK1 is always highly expressed in multiple cancers especially in glioma. Univariable and Multivariate proportional hazard Cox analysis showed that PLK1 was a prognostic biomarker for glioma. Simultaneously, highly expressed PLK1 is significantly related to prognosis, histological and genetic features in glioma by analyzing public databases. In addition, the enrichment analysis suggested that PLK1 might related to “immune response”, “cell cycle”, “DNA replication”, and “mismatch repair” in glioma. Immune infiltration analysis demonstrated that highly expressed PLK1 inhibited M1 macrophages infiltration to glioblastoma immune microenvironment by Quantiseq and Xcell databases and negatively related to some chemokines and marker genes of M1 macrophages in glioblastoma. Subsequent experiments confirmed that PLK1 knockdown inhibited the proliferation of glioma cells but increased the M1 macrophages infiltration and polarization. Furthermore, in glioma xenograft mouse models, we showed that inhibiting PLK1 blocked tumor proliferation and increased the M1 macrophages infiltration. Finally, PLK1 methylation analysis and lncRNA-miRNA network revealed the potential mechanism of abnormal PLK1 expression in glioma.

**Conclusions:**

PLK1 inhibits M1 macrophages infiltration into glioma immune microenvironment and is a potential biomarker for glioma.

## 1 Introduction

One of the most striking features of cancer cells is that aberrant cell cycle leads to their uncontrolled cell proliferation, which is usually caused by aberrant expression of the cell cycle-related genes (1). In recent years, more and more cell cycle-related genes were emerging as the candidate biomarkers for early diagnosis and potential therapeutic targets in cancers (2, 3).

Polo-like kinase (PLK) family has aroused our research interests because of its close relationship with cell cycle (4–6). PLKs belong to the serine/threonine kinase family that play differentiated and critical roles as key cell cycle regulators in tumor genesis and development (7, 8). PLKs are widely distributed in eukaryotic cells and the human PLKs family consists of five members, including PLK1, PLK2, PLK3, PLK4, and PLK5 (9).

Polo-like kinase1 (PLK1) is a highly conservative serine/threonine kinase widely found in eukaryotic cells and plays crucial roles in the cell cycle process (10). PLK1 is characterized by C-terminal serine domain (7), which can regulate N-terminal serine/serine kinase domain, mediates protein interaction and intracellular localization. PLK1 is also responsible for a wide range of cellular functions. It plays an important role in centriole maturation (11–13), Golgi disintegration (14), spindle assembling and function (15, 16), kinetochore function (17, 18), centromere assembling (19) and cytokinesis (20). It also facilitates DNA replication (21), mitotic entry (22), separation of sister chromatid (23), chromosome condensation (24) and APC/C activity (25). It has been reported that PLK1 is frequently over-expressed in numerous cancers (such as esophageal cancer, colon cancer, breast cancer, non-small cell lung cancer, endometrial cancer, etc.), and facilitates the occurrence and progress of these cancers acting as an oncogene (26). Although there has been a mass of researches on the roles of PLK1 in cancer about cell cycle, few analyses of PLK1 about tumor immunity have been conducted. To remedy this deficiency, we conducted an analysis of PLK1 in glioma immune regulation.

In this present study, we have comprehensively explored the PLK1 expression and its relationship with the prognosis of tumor patients in glioma across some datasets. And 100 glioma samples collected from surgeries were obtained to further validate the correlation between PLK1 mRNA expression and glioma grades or prognosis. Besides, the hypothesis that PLK1 is closely related to genetic alterations, immune, and cell cycle in tumors was also supported by KEGG and GO enrichment analysis of its related genes in glioma. Then, it was confirmed that PLK1 might be related to the tumor immunity of glioma by M1-like macrophages infiltration and polarization assays and intracranial xenograft mouse models. Some experiments such as flow cytometry and EdU also confirmed that aberrant expression of PLK1 lead to the occurrence and progression of glioma by regulating the cell cycle. In addition, we also explored the potential mechanisms of aberrant expression of PLK1 by analyzing PLK1 methylation and ceRNA network.

## 2 Materials and methods

### 2.1 Experimental methods

#### 2.1.1 RNA-seq of glioma samples

RNA-Seq data (lllumina HiSeq X Ten, Novogene) and corresponding pathological and clinical data of external 100 glioma samples were obtained to further validate the correlation between PLK1 mRNA expression and glioma grades or prognosis. All human glioma samples were taken from patients undergoing surgery at the First Affiliated Hospital of Zhengzhou University (**Supplementary Table 1**). The inclusion criteria were as follows: a. adult patients (>18 years) surgically treated and pathologically diagnosed primary WHO II-IV infiltrative gliomas, b. availability of clinical data. The exclusion criteria were as follows: a. incomplete survival data due to loss of follow up, b. recurrent glioma. Tissue samples were graded by neuropathologists according to the 2016 World Health Organization (WHO) standards and stored in liquid nitrogen. Glioma specimens were divided into LGG (33 cases) and HGG (67 cases). Our research was approved by the Human Scientific Ethics Committee of the First Affiliated Hospital of Zhengzhou University (FAHZZU) (Ethics approval: No. 2019-KY-176). All procedures performed in studies involving human participants were in accordance with the ethical standards of the institutional and national research committee and with the 1975 Helsinki declaration and its later amendments or comparable ethical standards. An informed consent was obtained from all individual participants included in the study.

#### 2.1.2 Cell culture and transfection

Human astrocyte, human glioblastoma cell lines (U87, U251, LN229, A172 and B19) and human THP-1 monocytes were obtained from ATCC (the American Type Culture Collection, Manassas, VA, USA). The human astrocyte was grown in Astrocyte Medium (AM) (ScienCell, USA) medium supplemented with 10% fetal bovine serum (Thermo Fisher Scientific, USA). Human glioblastoma cell lines (U87, U251, LN229, A172 and B19) and murine glioblastoma GL261 cells were grown in DMEM medium supplemented with 10% fetal bovine serum (Thermo Fisher Scientific, USA). THP-1 monocytes are maintained in culture in RPMI-1640 medium with 10% fetal bovine serum (Thermo Fisher Scientific, USA). Cell lines were tested using the ATCC cell line authentication service and routinely tested for Mycoplasma. All cells have been growing at 37°C in a humidified atmosphere (95% humidity) with 5% CO_2_ (27).

In knockdown experiment, human glioblastoma cells (U87 and LN229) were treated with PLK1-siRNA (GenePharma, Shanghai, China) by using Lipofectamine RNAimax (Thermo Fisher Scientific, USA) according to the manufacturer’s instructions. PLK1 siRNA 1#: 5’-CGAUACUACCUACGGCAAATT-3’; PLK1 siRNA 2#: 5’-CGAGGUGCUGAGCAAGAAATT-3’.

#### 2.1.3 RNA isolation and quantitative real-time PCR

The expression of mRNA in the cancer cell lines was detected by qRT-PCR. The total RNA of the cells was extracted using the TRIzol (Thermo Fisher Scientific, USA). The mRNA (1 µg) was reverse transcribed into cDNA by the reverse transcription kit (Promega, USA). The expression status of mRNA was measured on ABI QuantStudio 3 using GoTaq^®^ qPCR Master Mix (Promega, USA). GAPDH was selected as the loading control for mRNA expression analyses. cDNA product (2 µl) was used as template in a 20 µl PCR system containing 10 µl of GoTaq^®^ qPCR Master Mix and 2 µl of each primer. All reactions were performed in duplicate. Amplification protocols were as follows: 95°C for 10 min; 44 cycles of 95°C/10 s, 58°C/10 s, and 60°C/10 s. The primer sequences were as following: PLK1 Forward: 5’-TGACTCAACACGCCTCATCC-3’, Reverse: 5’-GCTCGCTCATGTAATTGCGG-3’. GAPDH Forward: 5ʹ-GGAGCGAGATCCCTCCAAAAT-3ʹ, Reverse: 5ʹ-GGCTGTTGTCATACTTCTCATGG-3ʹ. CCL2 Forward: AGCTTGTCTCAACCCCGCATC, Reverse: CCTTCAGGAACAGCCACCAATA. CCL5 Forward: CAGACCACGCAAGGAGTTCA, Reverse: CTTCCACCTTGGAGCACTGT. CCL7 Forward: TTGCTCAGCCAGTTGGGATTA, Reverse: TGGCTACTGGTGGTCCTTCT. CCL8 Forward: TGTCCCAAGGAAGCTGTGAT, Reverse: TGGAATCCCTGACCCATCTCT. CX3CL1 Forward: ACCACGGTGTGACGAAATG, Reverse: TGTTGATAGTGGATGAGCAAAGC. NOS2 Forward: CGCATGACCTTGGTGTTTGG, Reverse: CATAGACCTTGGGCTTGCCA. CD86 Forward: CTGCTCATCTATACACGGTTACC, Reverse: GGAAACGTCGTACAGTTCTGTG. CRR7 Forward: AGGAGAAGAAGGGTGCATTCG, Reverse: CGTCTTCCGTCACAAACTGC. Data were analyzed using the relative standard curve method and normalized to GAPDH (28, 29).

#### 2.1.4 EdU assay

EdU assay was used to examine cell proliferation. Glioma cell lines in the logarithmic growth phase were seeded into 96-well plates at a density of 2×10³- 4×10⁴. After 24 h, the adherent cells to the wells were transfected. Five parallel wells were set up for each group. Cells in each well after transfection for 48 h were cultured with 100 μL EdU medium (RIBOBIO, China) for 2 h and fixed with 100 μL of cell fixation solution (PBS containing 4% polyformaldehyde) for 30 min at room temperature. Subsequently, the cells were incubated with 2 mg/mL glycine (Solarbio, China) for 5 min, rinsed with 100 μL of PBS containing 0.5% TritonX-100 (RIBOBIO, China) for 10 min, and stained using 1 × Apollo staining reaction solution (RIBOBIO, China) for 30 min in conditions devoid of light. Next, the cells were reacted with 100 μL of the 1 × Hoechst 33342 reaction solution (RIBOBIO, China) for 30 min and sealed with 100 μL of the anti-fluorescence quenching agent. Six to ten fields of view were randomly selected for each well and photographed under a fluorescence microscope.

#### 2.1.5 Flow cytometry

Cell cycle arrest was analyzed by flow cytometry. The collected cells were washed with precooled 1×PBS for 3 times, and the supernatant was discarded after cell precipitation by centrifugation. After resuspend the cells with 0.5 mL 1×PBS, 3.5 mL 70% ethanol precooled was added quickly, beaten evenly, and stored overnight at 4°C. The supernatant of cells fixed by centrifugal ethanol was discarded, and then washed with 1×PBS for 3 times to remove the residual ethanol. The cells were resuspended with 1 mL Pi/Triton X-100 staining solution containing 0.2 mg RNase A (20 μg Pi/0.1% Triton X-100) and then stained at 37°C for 15 min. Finally, the cell cycle was measured by flow cytometry (Beckman Coulter, USA).

#### 2.1.6 Induced M1-like macrophages and M1-like macrophages infiltration assay

THP-1 cells were differentiated into M0 macrophages by 48 h incubation with 150 nM phorbol 12-myristate 13-acetate (PMA; P6741, Solarbio, China). Then, M0 macrophages were polarized to M1 macrophages by incubation with 20 ng/ml of IFN-γ (P00028, Solarbio, China) and 10 pg/ml of LPS (L8880, Solarbio, China) for 48 h.

M1 macrophages infiltration assays were applied through seeding 1.0×10^5^ M1 macrophages in the upper chamber of a transwell plate for 48 h (size 5mm, Corning, NY, USA). In bottom plate, U87/LN229 cells (1.0×10^5^) were cultured in DMEM. After incubation for 48 h, the cells in the upper chamber were fixed with 4% paraformaldehyde for 10 min and stained with 0.1% crystal violet for 10s. The infiltrated M1 macrophages were counted in three randomly selected fields from each membrane and each experiment was performed three times.

#### 2.1.7 Co-culture assay of macrophages and glioma cells

THP-1 cells were differentiated into M0 macrophages by 48 h incubation with 150 nM phorbol 12-myristate 13-acetate (PMA; P6741, Solarbio, China). For co-culture experiments, M0 macrophages were pre-inoculated in the 6-well plates (5.0×10^5^ cells per plate) and U87/LN229 cells (1.0×10^5^ cells per plate) were inoculated into the upper insert. After co-culture for 48 h, M0 macrophages were collected for the qRT-PCR to detect the effect of U87/LN229 cells on M0 macrophages polarization. All groups were repeated independently three times.

#### 2.1.8 Mouse model of glioma

Female C57BL/6 wild-type mice (4 weeks old, 16-20g) were used in our study. Five animals were housed per cage. All animals were maintained at controlled temperature (22 ± 2°C) and humidity (60–70%), under a 12 h light-dark cycle. All animals with regular chow and filtered water ad libitum. The mice used in our study were supplied by the Beijing HFK Bio-Technology (Beijing, China). All the experimental procedures were approved by the Animal Ethical and Welfare Committee of ZhengZhou University.

To create the intracranial tumor model, GL261 cells were infected with luciferase lentivirus (Genechem, China). Ketamine and xylazine (75 and 7.5 mg/kg, respectively) were injected to anesthetize the C57BL/6 mice. Then, the cells were injected with a 10-μl Hamilton microsyringe. An infusion pump was utilized to control the infusion rate at 1 μl/min × 2 min (in a total of 2 × 10^5^ cells per mouse). The injection site was selected at a depth of 3.0 mm in the right striatum of C57BL/6 wild-type mice (coordinates of bregma: 2.0 mm laterally). Standardized operations were adopted throughout the surgical procedures to avoid technical differences. The animals were randomly divided into control group (vehicle) and treatment group (PLK1 inhibitor volasertib). Starting on day 7 after tumor cell implantation, PLK1 inhibitor volasertib (20 mg/kg, i.p.) was given to the treated groups on twice a week for 3 weeks.

To acquire tumor growth status in live animals of different treatment groups by bioluminescent imaging on days 7, 14, 21, and 28, the mice were anesthetized and injected intraperitoneally with D-luciferin (150 mg/kg, beetle luciferin, potassium salt, E1605, Promega) 15 min prior to imaging with the IVIS imaging system (perkinelmer, USA) for 10-120 s. Four weeks after implantation, three animals per group were sacrificed and brain samples were collected for hematoxylin-eosin (HE) staining and immunofluorescence staining. The remaining mice in each group were used for bioluminescent imaging and survival analysis.

#### 2.1.9 Hematoxylin-eosin staining and immunofluorescence staining

After four weeks of tumor injection, the mice were euthanized and their brains were surgically removed and fixed in 10% neutral buffered formalin.

For HE staining, the 5 μm slides were deparaffinized and brought through a graded ethanol series to dH_2_O before the nuclei were stained with hematoxylin; the sections were then rinsed in running tap water and stained with eosin before being dehydrated and mounted. Pictures were taken using an Olympus upright BX53 microscope (Olympus). CellSens Entry software equipped with a digital CCD camera (Olympus DP22) was used.

For IF staining, Paraffin-embedded sections containing mice brains were deparaffinized, rehydrated, and subjected to antigen retrieval for staining of the M1 macrophage marker iNOS (CST, 13120). The processed brain tumor sections were incubated with primary antibody anti-iNOS (1:500) overnight at 4°C. Then, the sections were washed with PBS and followed by incubation with FITC (492-520 nm) (ZSGB-BIO, ZF-0311, China) for staining at room temperature for 60 min. Excess antibody was washed out with TBS, sections were counterstained with DAPI at room temperature for 5 min. All pictures were taken using an Olympus upright BX53 microscope (Olympus).

### 2.2 Bioinformatic analyses

#### 2.2.1 HPA: the human protein atlas

The Human Protein Atlas (HPA) (https://www.proteinatlas.org/) is a Swedish project launched in 2003 to map all human protein in cells, tissues and organs by integrating various omics technologies, including antibody-based imaging, mass spectrum-based protein omics, transcriptomics and systems biology (30–32).

The HPA online website was used to analyze PLK1 in “Tissue Atlas”, “Single Cell Type Atlas” and “Cell In Atlas” module. The expression data of PLK1 mRNA in different human normal tissues and tumor/non-tumor cells were obtained. The row data source was TMM normalized. Normalized eXpression (NX), the resulting transcript expression values, were calculated for each gene in every sample. Online website (https://www.proteinatlas.org/about/assays+annotation) showed the detailed information.

#### 2.2.2 The oncomine database

The expression levels of PLK1 gene in different tumor types were investigated in Oncomine (https://www.oncomine.org/resource/login.html) (**Supplementary Table 2**). The parameters were shown below: P-value<0.001, FC (fold change)>1.5 and gene rank is all (33, 34).

#### 2.2.3 The online platform: SangerBox

We obtained a simulation map of the subcellular localization of the PLK1 protein using the SangerBox online tool (http://SangerBox.com/Tool). The COX_OS (overall survival), COX_DFS (disease free survival), and Neoantigen analysis of PLK1 for different tumors was analyzed on SangerBox portal. Immune cells infiltration analysis in glioma was also conducted by Sangerbox. In addition, the SangerBox online tool can also perform PLK1-related s KEGG and GO enrichment analysis.

#### 2.2.4 GEPIA2: gene expression profiling interactive analysis 2.0

The online website: Gene Expression Profiling Interactive Analysis 2.0 (GEPIA2) (http://gepia2.cancer-pku.cn/#index) is an interactive web that includes 9,736 tumors and 8,587 normal samples from TCGA and the GTEx databases, which analyses the RNA sequencing expression (35, 36). In this present study, GEPIA2 was also used to conduct survival curves, including overall and disease-free survival in 33 different cancer types. The correlation analysis of gene expression was conducted using the given TCGA expression dataset. PLK1 positively related genes in pan-cancer were also obtained in GEPIA2 database (**Supplementary Table 3**). The correlation coefficient was determined by Spearman’s statistical method.

#### 2.2.5 Chinese glioma genome atlas and the cancer genome atlas datasets

The RNA-seq data and clinical information of CGGA-325 (http://www.cgga.org.cn/download.jsp) are shown in **Supplementary Table 4**; The RNA-seq data and clinical information of CGGA-693 (http://www.cgga.org.cn/download.jsp) are shown in **Supplementary Table 5**; The RNA-seq data and clinical information of TCGA (https://www.cancer.gov/about-nci/organization/ccg/research/structural-genomics/tcga) are shown in **Supplementary Table 6**.

#### 2.2.6 The STRING database

We entered the STRING website (https://string-db.org/) using the query of a single protein name (“PLK1”) and organism (“Homo sapiens”). Next, we optioned the threshold as following: minimum required interaction score [“Low confidence (0.150)”], meaning of network edges (“evidence”), max number of interactors to show (“no more than 50 interactors” in 1st shell) and active interaction sources (“experiments”). At last, the PLK1-binding proteins and related PPI network that had been experimentally confirmed were obtained (37, 38).

#### 2.2.7 The GEO databases

GSE67102 (https://www.ncbi.nlm.nih.gov/geo/query/acc.cgi?acc=GSE67102) and GSE46856 (https://www.ncbi.nlm.nih.gov/geo/query/acc.cgi?acc=GSE46856) databases were used to analyze the biological functions of PLK1 related genes. The differentially expressed genes were obtained after treating with inhibitors of PLK1 in GSE67102 and GSE46856 databases. Then KEGG and GO-BP enrichment analyses of PLK1 related genes was analyzed and mapped.

#### 2.2.8 The cBioportal website

After logging into the cBioPortal website (https://www.cbioportal.org/), we chose the “TCGA Pan Cancer Atlas Studies” in the “Quick select” section and entered “PLK1” to query of the gene alteration characteristics of PLK1. We obtained the results of the alteration frequency, mutation type and copy number alteration (CNA) among all TCGA tumors in the “Cancer Types Summary” module. The information on the mutations site of PLK1 can be displayed in the protein structure diagram or 3D structure through the “Mutilations” module (39, 40).

#### 2.2.9 The web portal TISIDB

TISIDB (http://cis.hku.hk/TISIDB/index.php) is an online website that integrates tumor and immune system interactions across multiple heterogeneous data types (41). We got the relationship between the abundance of tumor-infiltrating lymphocytes (TILs) and the PLK1 mRNA levels in this study. We also got the relationship between the immune cell chemokines levels and the PLK1 mRNA levels in TISIDB.

#### 2.2.10 Genomic identification of significant targets in cancer 2.0

Databases of somatic mutations and somatic copy number alternations (CNAs) were obtained from TCGA datasets. CNAs correlated with PLK1 mRNA levels, and the threshold copy number at alteration peaks were analyzed by GISTIC 2.0 (https://cloud.genepattern.org/). The patients were divided into the first 25% PLK1-low (n=170) and the last 25% PLK1-high (n=170) groups according to the expression value of PLK1. The maftools package was also used in R software (https://www.r-project.org/) to download and visualize the somatic mutations.

#### 2.2.11 The UALCAN database

The UALCAN portal (http://ualcan.path.uab.edu/analysis-prot.html), an interactive web resource, is usually used to analyze cancer Omics data (42). In present study, we used it to compare the differential expressions of PLK1 protein in a specific tumor and its corresponding normal tissue and the methylation levels of the PLK1 promoter region between some primary tumors and normal tissues.

#### 2.2.12 Single-cell RNA analysis

We processed the single-cell data expression matrix with the R package Seurat. We employed “NormalizeData” to normalize the gene expression data, followed by utilizing “FindVariableGenes” to identify 2,000 highly variable genes (HVGs). Then, we performed principal component analysis (PCA) with “RunPCA”, built a K-nearest neighbor graph *via* the “FindNeighbors” function, and grouped cells in the highest resolution with “FindClusters.” Finally, “TSNE” was used for visualization, and we performed a “Single R” R package for cell annotation. “VlnPlot” were used to visualize PLK1 expression. Single-cell pseudotime trajectories were reconstructed with package “Monocle”. GSE84465 (https://www.ncbi.nlm.nih.gov/geo/query/acc.cgi?acc=GSE84465) was used to conducted Single-Cell RNA Analysis in this study.

#### 2.2.13 Statistical analysis

Through the online databases mentioned above, the statistical analysis was automatically computed in this study. These results were considered as statistically significant at *P<0.05, **P<0.01, ***P<0.001 and ****P<0.0001.

## 3 Results

### 3.1 The expression pattern of PLK1 mRNA in pan-cancer

**Figure 1:**
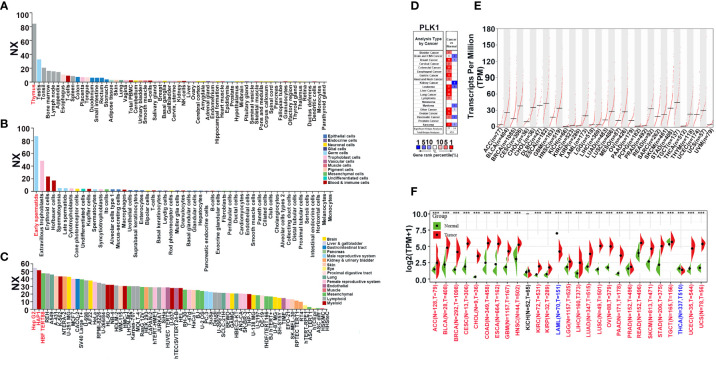
The expression pattern of PLK1 mRNA in pan-cancer. **(A)**. The expression status of the PLK1 mRNA in different human tissues were analyzed through HPA. **(B)**. The expression status of the PLK1 mRNA in different human cells were analyzed through HPA. **(C)**. The expression status of the PLK1 mRNA in different tumor cell lines were analyzed through HPA. **(D)**. Increased or decreased PLK1 mRNA in datasets of different cancers compared with normal tissues in the Oncomine database (*P*<0.001, FC>1.5, gene rank=all). **(E)**. PLK1 mRNA expression levels in different tumor types from TCGA database were determined by GEPIA2. **(F)**. PLK1 mRNA expression levels in different tumor and normal tissues from TCGA and GTEx database were determined by SangerBox (***P<0.001).

The flowchart of this study is shown in **Supplementary Figure 1**. The Normalized eXpression (NX) levels of PLK1 were analyzed in various tumor tissues and their corresponding adjacent normal tissues as well as various tumor cells and the corresponding non-tumor cells in the Human Protein Atlas (HPA) database. PLK1 mRNA expression levels were higher in the normal human thymus, testis, and tonsil (NX>20; **Figure 1A**). In most other normal human tissues, PLK1 mRNA expression levels were detectable but low (NX<20) (**Figure 1A**). PLK1 mRNA expression levels were higher in the early spermatids, extravillous trophoblasts and erythroid cells (NX>20; **Figure 1B**). In most other normal human cells, PLK1 mRNA expression levels were detectable but low (NX<20) (**Figure 1B**). In human tumor cell lines, the expression level of PLK1 mRNA was the most abundant in human hepatocellular carcinomas cell lines (Hep G2), followed by human leukemia cell lines (HAP1) (**Figure 1C**). Moreover, in order to learn the differences of PLK1 mRNA expression in cancer and normal tissues, we analyzed the expression levels of PLK1 mRNA in different tumor tissues and normal tissues through the Oncomine website. The results suggested that the expression levels of PLK1 mRNA were higher in bladder, brain and CNS (Central Nervous System), colorectal, gastric, breast, esophageal, cervical, head and neck, ovarian, lung, liver, pancreatic cancer and lymphoma, sarcoma, leukemia compared to the normal tissues (**Figure 1D**). The integrated conditions of PLK1 expression in various cancers were collected in **Supplementary Table 2**. To further learn the PLK1 mRNA expression condition in different cancers, we tested the PLK1 mRNA expression across the RNA-seq data of a variety of malignancies in TCGA. There displayed the mRNA expression levels of PLK1 in all TCGA tumors. There is a significant difference of PLK1 mRNA expression levels among TCGA tumors (**Figure 1E**). Moreover, we further analyzed the differential expressions of PLK1 mRNA between tumor tissues and normal tissues using the TCGA and GTEx databases with SangerBox. The expression of PLK1 mRNA was statistically higher in 24 cancers: adrenocortical carcinoma(ACC), bladder rothelial carcinoma (BLCA), breast invasive carcinoma(BRCA), cervical squamous cell carcinoma and endocervical adenocarcinoma (CESC), cholangiocarcinoma (CHOL), colon adenocarcinoma (COAD), esophageal carcinoma (ESCA), glioblastoma multiforme (GBM), head and neck cancer (HNSC), kidney renal clear cell carcinoma (KIRC), kidney renal papillary cell carcinoma (KIRP), brain lower grade glioma (LGG), liver hepatocellular carcinoma (LIHC), lung adenocarcinoma (LUAD), lung squamous cell carcinoma (LUSC), ovarian serous cystadenocarcinoma (OV), pancreatic adenocarcinoma (PAAD), prostate adenocarcinoma (PRAD), rectum adenocarcinoma (READ), skin cutaneous melanoma (SKCM), stomach adenocarcinoma (STAD), testicular germ cell tumors (TGCT), uterine corpus endometrial carcinoma (UCEC), and uterine carcinosarcoma (UCS). Whereas, the PLK1 mRNA expressions were lower in acute myeloid leukemia (LAML) and thyroid carcinoma (THCA) (**Figure 1F**).

Then, since our team specializes in glioma, we focused on the relationship between PLK1 mRNA levels and prognosis of patients with glioma. Next, we analyzed PLK1 mRNA expression levels in different WHO grades and histologic classifications of gliomas. The results showed that PLK1 expression levels were positively associated with glioma grades CGGA and TCGA databases (**Supplementary Figure 2A**). And the receiver operating characteristic (ROC) curve verified that PLK1 could be an effective factor for predicting WHO grades of glioma (**Supplementary Figure 2B**). Moreover, the expression levels of PLK1 mRNA correlated with the histologic classification of gliomas (**Supplementary Figure 2C**). Isocitrate dehydrogenase (IDH) mutations and chromosomal 1p/19q codeletions are associated with better survival outcomes of glioma patients. Furthermore, promoter methylation status of the O6-methylguanine DNA methyltransferase (MGMT) is a prognostic indicator of the clinical response to treatment of glioblastoma patients with temozolomide (TMZ). Then, we explored the relationship between PLK1 mRNA expression and the status of IDH gene mutations, 1p/19q codeletion, and MGMT promoter methylation. Analysis of the CGGA-325, CGGA-693 datasets and TCGA datasets showed that PLK1 mRNA levels in the glioma patients with wild-type IDH and chromosomal 1p/19q non-codeletion were significantly higher (**Supplementary Figures 2D-E**), however, there is no significantly difference in the glioma patients with MGMT promoter methylation in CGGA datasets (**Supplementary Figure 2F**).

In conclusion, the above results suggested that PLK1 mRNA levels were upregulated in several tumors. Furthermore, PLK1 expression levels correlated with the grades, the histologic classification, and clinical features of gliomas.

### 3.2 The expression pattern of PLK1 protein in pan-cancer

**Figure 2:**
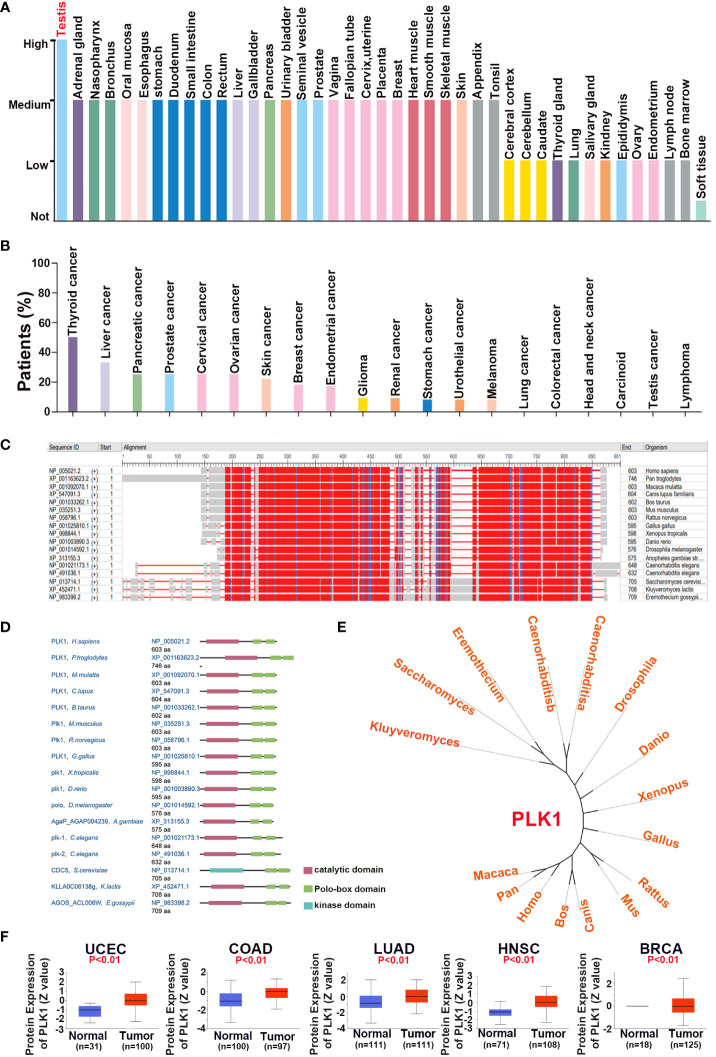
The expression pattern of PLK1 protein in pan-cancer. The expression status of the PLK1 protein in different human normal **(A)** and cancer **(B)** tissues were analyzed through HPA. **(C)**. The conserved analysis of PLK1 amino acid among different species by NCBI. **(D)**. The conserved analysis of PLK1 domain among different species by NCBI. **(E)**. The phylogenetic tree data presents the evolutionary relationship of the PLK1 protein across different species. **(F)**. The differential expression of PLK1 protein in normal tissues and tumors in UCEC, COAD, LUAD, HNSC and BRCA across the CPTAC database of UALCAN online tool.

In this part, our aim was to explore the oncogenic roles of the human PLK1 protein. We investigated the expression characteristics of the PLK1 protein in 41 different normal tissues and various cancers using the HPA database, respectively. The analysis results appeared that the expression of PLK1 protein in human normal tissues was highest in testis, while the expression in other tissues was low or moderate (**Figure 2A**). Whereas, PLK1 protein in human cancer tissues was highest in thyroid cancer, while the expression in glioma tissues was moderate (**Figure 2B**). The conserved analysis of PLK1 protein among different species showed that the amino acid sequence and domain of PLK1 protein is conserved among different species (**Figures 2C, D**). The phylogenetic tree figure presented the evolutionary relationship of the PLK1 proteins among various species (**Figure 2E**). The CPTAC database of UALCAN online tool was used to explore the differential expressions of PLK1 proteins in tumor and normal tissues. The analysis results appeared that the PLK1 protein expression levels in UCEC, COAD, LUAD, HNSC and BRCA were overexpressed than that in normal tissues (**Figure 2F**).

These results suggested that the domain of PLK1 protein is conserved between different species, and PLK1 protein may play a carcinogenic role in some tumors, such as uterine corpus endometrial carcinoma, colon cancer and lung adenocarcinoma.

### 3.3 PLK1 expression is associated with the prognosis in pan-cancer including gliomas

**Figure 3:**
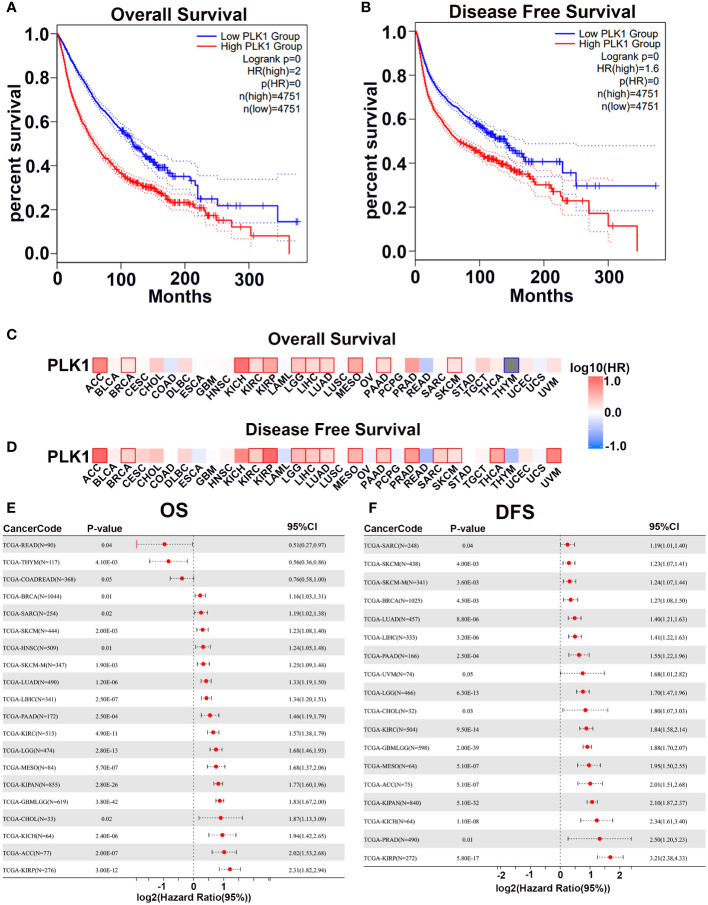
The prognostic potential of PLK1 mRNA expression levels in pan-caner. The overall survival **(A)** and disease-free survival **(B)** analyses of pan-cancer in TCGA about PLK1 mRNA level using GEPIA2. The overall survival **(C)** and disease-free survival **(D)** analyses of different tumors in TCGA about PLK1 mRNA level using GEPIA2. **(E)**. Relationship between PLK1 mRNA level and Cox-OS of different cancers in TCGA datasets using SangerBox. **(F)**. Relationship between PLK1 mRNA level and Cox-DFS of different cancers in TCGA datasets using SangerBox.

First, we analyzed the correlation between PLK1 mRNA levels and prognosis in pan-cancer by GEPIA2 with TCGA database and plotted survival curves for overall survival (OS) and disease-free survival (DFS) respectively. The results showed that higher PLK1 mRNA levels were statistically related to poorer OS and DFS in pan-cancer (**Figures 3A, B**). Then we further analyzed the correlation between the expression levels of PLK1 mRNA and prognosis in specific tumor types. The analysis appeared that higher PLK1 mRNA expression levels were statistically related to the poorer OS of adrenocortical carcinoma (ACC), breast invasive carcinoma (BRCA), kidney chromophobe (KICH), kidney renal clear cell carcinoma (KIRC), kidney renal papillary cell carcinoma (KIRP), brain lower grade glioma (LGG), liver hepatocellular carcinoma (LIHC), lung adenocarcinoma (LUAD), Mesothelioma (MESO), pancreatic adenocarcinoma (PAAD), skin cutaneous melanoma (SKCM) (**Figure 3C**), and poorer DFS of adrenocortical carcinoma (ACC), breast invasive carcinoma (BRCA), kidney renal clear cell carcinoma (KIRC), kidney renal papillary cell carcinoma (KIRP), brain lower grade glioma (LGG), liver hepatocellular carcinoma (LIHC), lung adenocarcinoma (LUAD), Mesothelioma (MESO), pancreatic adenocarcinoma (PAAD), prostate adenocarcinoma (PRAD), Sarcoma (SARC), skin cutaneous melanoma (SKCM), thyroid carcinoma (THCA), uveal melanoma (UVM) (**Figure 3D**).Cox regression analysis of the SangerBox database showed that PLK1 mRNA levels were associated with OS and DFS of patients with multiple cancers. The results showed that the high mRNA levels of PLK1 were associated with shorter OS in breast invasive carcinoma (BRCA), Sarcoma (SARC), skin cutaneous melanoma (SKCM), head and neck squamous cell carcinoma (HNSC), skin cutaneous melanoma-metastasis (SKCM-M), lung adenocarcinoma (LUAD), liver hepatocellular carcinoma (LIHC), pancreatic adenocarcinoma (PAAD), kidney renal clear cell carcinoma (KIRC), brain lower grade glioma (LGG), Mesothelioma (MESO), Pan-kidney cohort, (KIPAN), glioma (GBM+LGG), cholangiocarcinoma (CHOL), kidney chromophobe (KICH), adrenocortical carcinoma (ACC), and kidney renal papillary cell carcinoma (KIRP) and shorter DFS in Sarcoma (SARC), skin cutaneous melanoma (SKCM), skin cutaneous melanoma-metastasis (SKCM-M), breast invasive carcinoma (BRCA), lung adenocarcinoma (LUAD), liver hepatocellular carcinoma (LIHC), pancreatic adenocarcinoma (PAAD), uveal melanoma (UVM), brain lower grade glioma (LGG), cholangiocarcinoma (CHOL), kidney renal clear cell carcinoma (KIRC), glioma (GBM+LGG), Mesothelioma (MESO), adrenocortical carcinoma (ACC), kidney chromophobe (KICH), prostate adenocarcinoma (PRAD), and kidney renal papillary cell carcinoma (KIRP) (**Figures 3E, F**).

The correlation between PLK1 mRNA levels and the prognosis of patients with pan-glioma, LGG, and GBM was investigated using the CGGA and TCGA datasets. In the CGGA-325, CGGA-693, and TCGA datasets, high PLK1 mRNA levels were associated with shorter OS in pan-glioma and HGG patients (**Supplementary Figures 3A, C**). However, the relationship between PLK1 mRNA levels and OS in LGG patients was not statistically significant in any of the three datasets (**Supplementary Figure 3B**). And the ROC curve verified that PLK1 could be an effective factor for predicting pan-glioma in the CGGA-325, CGGA-693, and TCGA datasets (**Supplementary Figure 3D**). Moreover, high PLK1 mRNA levels were associated with shorter disease specific survival (DSS) and progression free survival (PFI) in pan-glioma and HGG patients in TCGA dataset (**Supplementary Figures 3E, F**). In addition, multiple Cox regression revealed grade, IDH mutations, 1p/19q codeletions, promoter methylation of MGMT, and PLK1 mRNA levels might be independent predictors of prognosis of glioma patients (**Supplementary Figure 4A**). Similarly, the nomogram showed similar results (**Supplementary Figure 4B**). Therefore, we next explored the relationship between these molecular indicators and the prognosis of patients with glioma. As shown in **Supplementary Figure 5A**, patients in PLK1-high group had poorer prognosis compared to those in PLK1-low group in both IDH mutated and non-mutated glioma patients in CGGA-325 and CGGA-693 datasets. As shown in **Supplementary Figure 5B**, patients in PLK1-high group had poorer prognosis compared to those in PLK1-low group only in 1p19q non-codeletion glioma patients in CGGA-325, CGGA-693, and TCGA datasets. As shown in **Supplementary Figure 5C**, patients in PLK1-high group had poorer prognosis compared to those in PLK1-low group both in MGMT promoter methylated and no-methylated glioma patients in CGGA-325, CGGA-693, and TCGA datasets. As shown in **Supplementary Figures 5D, E**, patients in PLK1-high group had poorer prognosis compared to those in PLK1-low group both in chemoradiotherapy and no-chemoradiotherapy glioma patients in CGGA-325 and CGGA-693 datasets.

Overall, the results demonstrated that the PLK1 mRNA levels were associated with the prognosis of multiple cancers. Moreover, higher PLK1 mRNA levels were associated with poorer prognosis of glioma patients.

### 3.4 Enrichment analysis of PLK1 related genes

**Figure 4:**
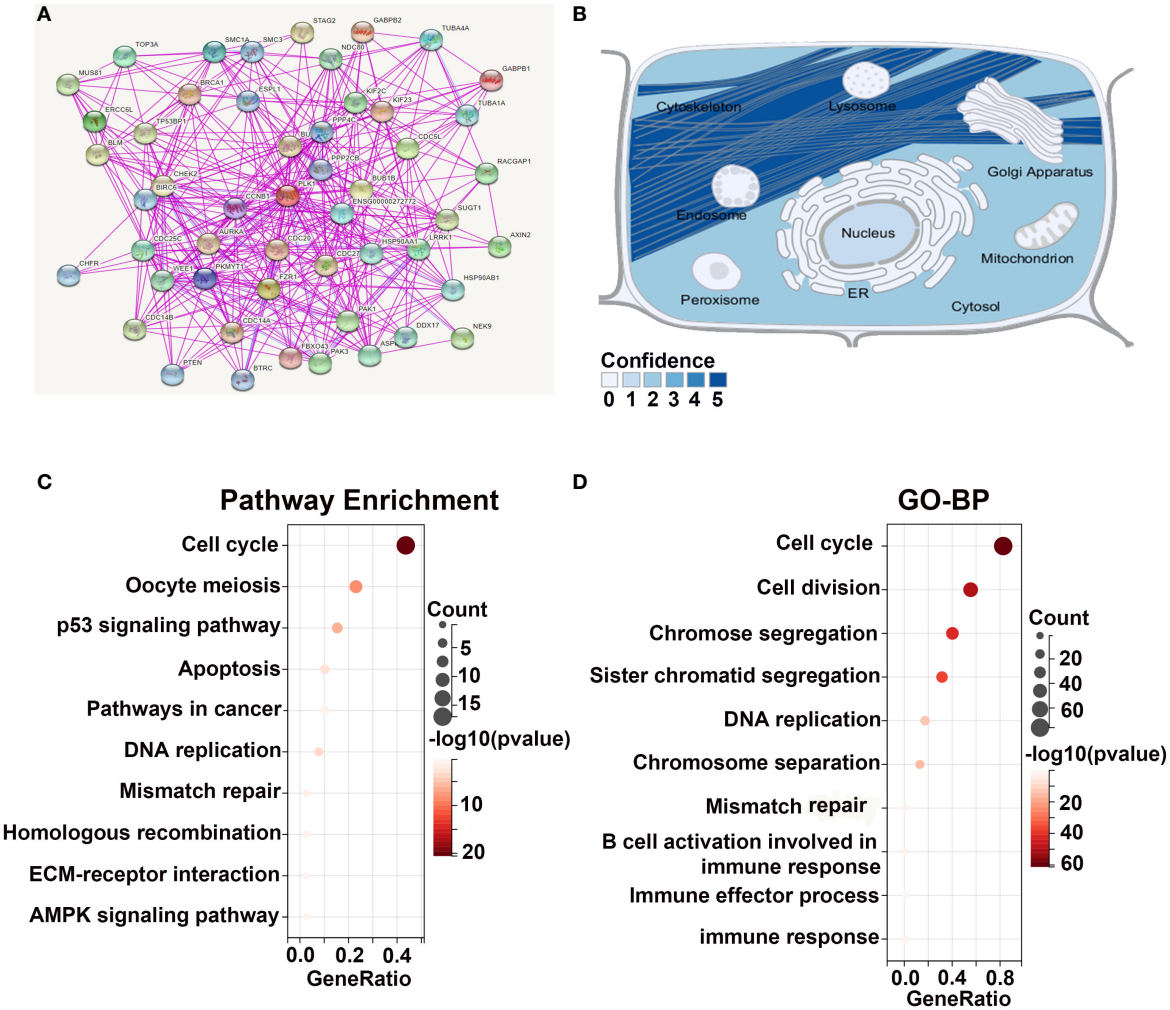
Enrichment analysis of PLK1-related genes. **(A)**. The experimentally determined PLK1-binding proteins were obtained using the STRING tool. **(B)**. The simulation of the intracellular localization of PLK1 protein. The KEGG **(C)** and GO-BP **(D)** enrichment analyses were applied with the top 100 positively genes correlated with PLK1 expression in pan-cancer.

In order to further explore the molecular mechanisms of PLK1 in tumorigenesis among pan-cancer, we mined PLK-binding proteins to conduct a protein-protein interaction network and the PLK1 expression-related genes to perform a battery of enrichment analyses. We obtained 50 PLK1-binding proteins with experimental verification based on the online website STRING. And the network diagram graphically showed the interactions of these proteins (**Figure 4A**). In order to determine the subcellular localization of the PLK1 proteins, we used the SangerBox database to investigate that the PLK1 proteins were mainly localized on the cytoskeleton of the cytoplasm (**Figure 4B**). Furthermore, we obtained a total of top 100 genes significantly positively correlated with PLK1 gene by GEPIA2 with TCGA database (**Supplementary Table 3**). Subsequently, we performed KEGG and GO-BP enrichment analyses using the top 100 positively correlated genes. The results of KEGG and GO-BP enrichment analyses are shown in **Figures 4C, D**. Our enrichment results showed that the top 100 genes were enriched not only in cell cycle-related pathways and terms but also in genetic alterations and immune related pathways and terms, such as “cell cycle”, “mismatch repair”, and “immune response” (**Figures 4C, D**).

In addition, we conducted enrichment analyses using PLK1 related genes in glioma. Analyses of PLK1 related genes in CGGA-325, TCGA and FAHZZU (The First Affiliated Hospital of Zhengzhou University) databases were performed using R package. Then, the heat map of PLK1-related genes was shown in **Supplementary Figures 6A–C**. And we performed KEGG and GO-BP enrichment analyses using the correlated genes (R>0.35 in CGGA-325; R>0.55 in TCGA; R>0.55 in FAHZZU) (**Supplementary Figures 6A–C**). Similarly, GSE67102 and GSE46856 databases were used to analyze the biological functions of PLK1 related genes. And the volcano plot, KEGG, GO-BP enrichment analyses of PLK1 related genes was analyzed and mapped in glioma by SangerBox portal (**Supplementary Figures 7A, B**). Like the results in pan-cancer, the results demonstrated that compared to glioma with low PLK1 expression, glioma with high PLK1 expression were enriched not only in classical carcinogenic signaling pathways and terms but also in cell cycle, genetic alterations, and immune related pathways and terms (**Supplementary Figures 6, 7**).

Therefore, based on these analysis results, we speculated that PLK1 might promote tumor genesis and development by affecting cell cycle, genetic alterations, and antitumor immune in pan-cancer, especially in glioma.

### 3.5 Alterations of PLK1 gene are associated with development and progression of pan-cancer including glioma

Enrichment analysis of PLK1 related genes showed that PLK1 might promote tumor genesis and development by affecting genetic alterations. Genetic alterations such as the mutations, deletions, or amplifications of oncogenes or tumor suppressor genes are associated with growth and progression of several tumors. Therefore, we first analyzed different types of alterations including mutations, structural variations, amplifications, and deep deletions in the *PLK1* gene in using the TCGA cancer datasets with the cBioPortal portal. Among the 32 tumor types, *PLK1* gene alteration frequency was the highest in UCEC cases (>5%), and the “mutation” type was dominant (**Supplementary Figure 8A**). What is noteworthy is that all MESO (~1.0% frequency) and PAAD (~0.5% frequency) cases with gene alteration are “amplification” type (**Supplementary Figure 8A**). In addition, we discovered that the most abundant mutation type of PLK1 was “missense mutation” in pan-cancer (**Supplementary Figure 8B**). R293H/C alteration in the Pkinase domain of PLK1 protein, which was discovered in 2 cases of COAD, 1 case of LUAD, 1 case of ESCA and 1 case of HNSC, can lead to missense mutation of the *PLK1* gene, translating from R (Arginine) to H (Histidine) or C (Cysteine) at the site 293 of PLK1 protein, and changing the structure of PLK1 protein subsequently (**Supplementary Figure 8B**). The genetic alteration effect of R293H/C was displayed in the 3D structure of PLK1 protein (**Supplementary Figure 8C**).

In addition, to determine whether PLK1 expression levels were associated with specific genomic characteristics in gliomas, we performed copy number variation (CNV) and somatic mutation analysis using the TCGA dataset. A distinct overall CNV profile emerged from the comparison of the PLK1-low (n = 170) versus the PLK1-high (n = 170) cluster (**Supplementary Figures 8D–F**). Co-deletion of 1p and 19q, a genomic hallmark of oligodendroglioma, more frequently appeared to be associated with the PLK1-low cluster (**Supplementary Figure 8D**). Amplification of chr7 and deletion of chr10, which are both common genomic events in GBM, frequently occurred in the PLK1-high cluster (**Supplementary Figure 8D**). The comparison of the CNV profiles in the PLK1-low (n = 170) and PLK1-high (n = 170) samples is shown in **Supplementary Figures 8E, F**. In PLK1-high group, frequently amplified genomic regions included oncogenic driver genes, such as EGFR (7p11.2), IK3C2B (1q32.1), PDGFRA (4q12), and CDK4 (12q14.1), whereas deleted regions contained tumor suppressor genes, including CDKN2A/CDKN2B (9p21.3), PARK7 (1p36.23), and PTEN (10q23.3). In PLK1-low samples, significant amplifications showed peaks in 7p11.2, 8q24.13, 12p13.3, and 19p13.3, whereas the frequently deleted genomic regions were 2q37.3, 9p21.3, 13q21.33, and 14q23.2. The PLK1-low group (n = 170) showed high frequency of somatic mutations in the IDH1 (75%), TP53 (36%), ATRX (32%), and CIC (28%) genes and the PLK1-high group (n = 170) showed high frequency of mutations in the TP53 (38%), TTN (33%), PTEN (31%), and EGFR (30%) genes (**Supplementary Figures 8G, H**).

Overall, these results showed *PLK1* gene mutations, amplifications, and deletions in multiple tumors and missense mutations were the most frequent type. Moreover, the glioma tissues showed distinct somatic mutations and CNVs based on the expression levels of PLK1. These results suggested that the alteration of *PLK1* gene might be a potential mechanism to lead to the occurrence and development of various tumors, especially glioma.

### 3.6 PLK1 expression is associated with the antitumor immunity in pan-cancer including glioma

Enrichment analysis of PLK1 related genes implied that PLK1 might promote tumor genesis and development by affecting antitumor immune in pan-cancer. Therefore, we also analysis the relationship between PLK1 expression and the tumor immune microenvironment (TIM) in pan-cancer, especially in glioma.

**Figure 5:**
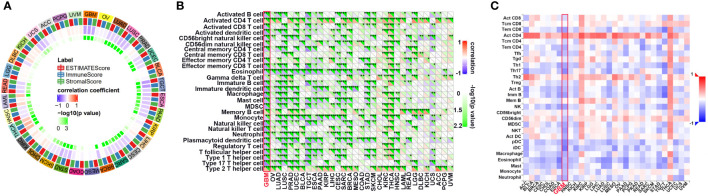
The relationship between the PLK1 mRNA levels and antitumor immune in pan-cancer. **(A)**. The correlations between ESTIMATE scores (ESTIMATE Score, Immune Score, and Stromal Score) and PLK1 mRNA levels were analyzed in various tumors by SangerBox portal. **(B)**. The relationship between PLK1 mRNA levels and immune cell infiltration levels was analyzed in various tumors by SangerBox. **(C)**. The relationship between PLK1 mRNA levels and abundance of tumor-infiltrating lymphocytes (TILs) using TISIDB. *P<0.05, **P<0.01, ***P<0.001, ****P<0.000.1.

**Figure 6:**
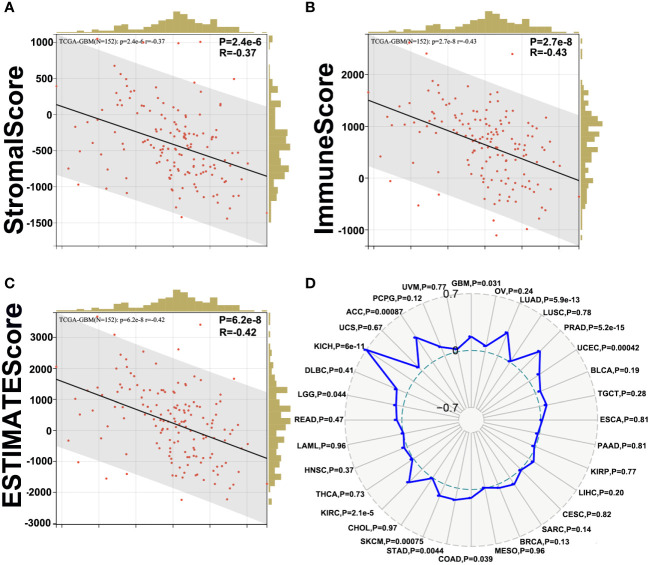
Correlation between PLK1 expression and ESTIMATE Score in glioma. The correlations between PLK1 mRNA level and Stromal Score (*P*=1.9e-8, R=-0.44) **(A)**, Immune Score (*P*=1.6e-6, R=-0.38) **(B)** and ESTIMATE Score (*P*=4.1e-8, R=-0.43) **(C)** of GBM in TCGA dataset. **(D)**. The relationship between PLK1 mRNA expression and TMB in multiple cancers.

First, we evaluated the correlation between ESTIMATE scores (ESTIMATE, immune, and stromal scores) and PLK1 mRNA levels in pan-cancer. Immune score reflects the proportion of infiltrated immune cells in the tumor tissues; stromal score reflects the proportion of stromal cells in the tumor tissues. ESTIMATE score is the sum of immune and stromal scores, and reflects the status of the tumor immune microenvironment and tumor purity. Our results demonstrated negative correlation between PLK1 mRNA levels and the ESTIMATE, immune, and stromal scores in glioblastoma multiforme (GBM), lung adenocarcinoma (LUAD), lung squamous cell carcinoma (LUSC), uterine corpus endometrial carcinoma (UCEC), testicular germ cell tumors (TGCT), esophageal carcinoma (ESCA), pancreatic adenocarcinoma (PAAD), cervical squamous cell carcinoma and endocervical adenocarcinoma (CESC), sarcoma (SARC), stomach adenocarcinoma (STAD), skin cutaneous melanoma (SKCM), head and neck squamous cell carcinoma (HNSC), and rectum adenocarcinoma (READ) (**Figure 5A**). This suggested that high PLK1 mRNA levels were associated with decreased infiltration of immune and stromal cells in these tumors. **Figure 6** showed the negative correlations between PLK1 mRNA levels and ESTIMATE score, Immune score and Stromal score in GBM, suggesting low immune pressure in PLK1 overexpressed patients (**Figures 6A–C**). Quksza et al. conceive a model to elucidate that low immune pressure induces high tumor heterogeneous and tumor mutational burden (TMB). We therefore hypothesized that this low immune pressure would induce tumors much more heterogeneous and TMB in PLK1 overexpressed patients (43), which was consistent with our analysis results in many tumors including glioma (**Figure 6D**).

**Figure 7:**
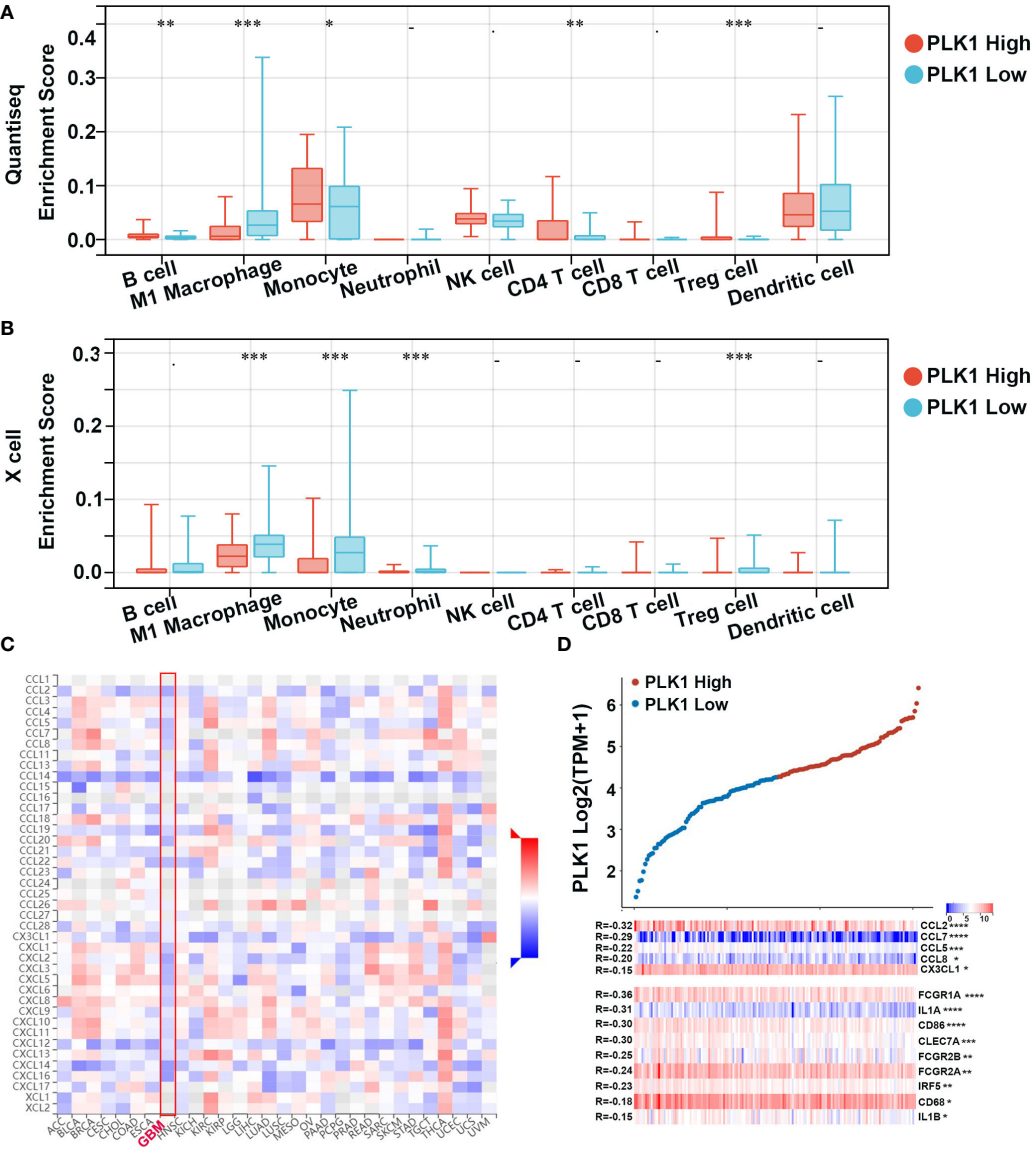
Correlation analysis between PLK1 expression and immune cell or chemokines in glioma. Comparison of immune cell infiltration between PLK1 low expression and PLK1 high expression groups in GBM by Quantiseq **(A)** and Xcell **(B)** in TCGA dataset. *P<0.05, **P<0.01, ***P<0.001, ****P<0.000.1. **(C)**. The relationship between PLK1 mRNA expression and chemokines in multiple cancers. **(D)**. The relationship between PLK1 mRNA expression and M1 macrophages-related chemokines/markers in GBM. *P<0.05, **P<0.01, ***P<0.001, ****P<0.000.1.

Tumor-infiltrating immune (TIIs) cells, as an important part of TIM, are usually related to the occurrence, progression, treatment, or metastasis of tumor. Moreover, many reports have claimed that tumor-infiltrating lymphocytes (TILs) are critical predictors of sentinel lymph node status and survival in cancers (44). Thus, we then used the online tool Sangerbox and TISIDB to analysis the relationship between abundance of TIIs/TILs and PLK1 mRNA levels. PLK1 mRNA levels showed negative correlation with multiple TIIs/TILs in GBM (**Figures 5B, C**). Interestingly, it is also significantly negative correlated between PLK1 mRNA levels and M1 macrophages infiltration levels in GBM in TCGA dataset using the Quantiseq and X cell (**Figures 7A, B**). The same trend was observed between the expression of PLK1 and macrophages chemokines in TCGA dataset in glioma (**Figures 7C, D**).

**Figure 8:**
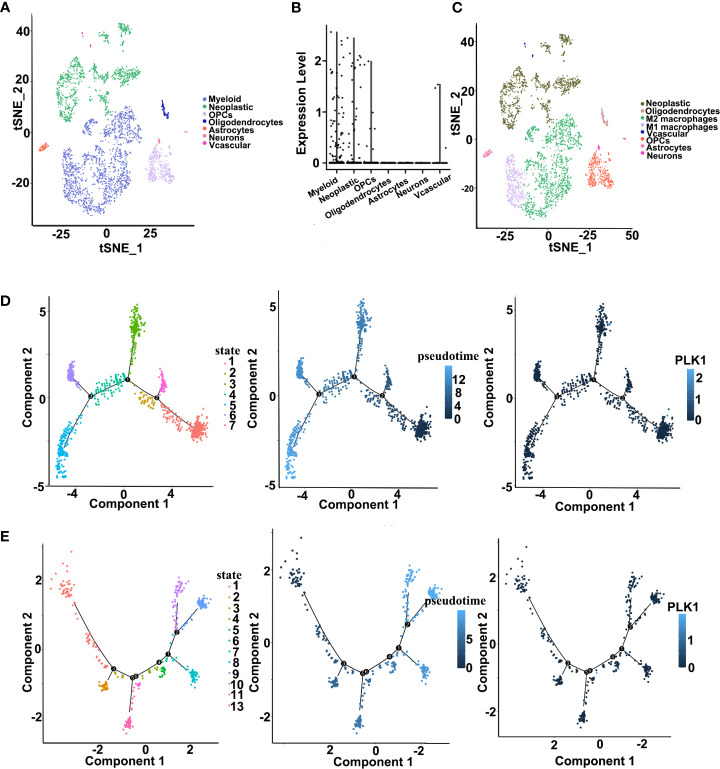
Single cell sequencing analysis for PLK1 in GBM. **(A)**. Cells were annotated into seven clusters and were annotated as myeloid cells, neoplastic cells, oligodendrocyte precursor cells, oligodendrocytes, astrocytes, neurons, and vascular cells. **(B)**. Violin plot of PLK1 expression distribution of seven cell clusters. **(C)**. Cells were annotated into eight clusters and were annotated as neoplastic cells, oligodendrocytes, M2-like macrophages, M1-like macrophages, vascular cells, oligodendrocyte precursor cells, astrocytes, and neurons. **(D)**. Single-cell trajectory analysis of neoplastic cells shows four main branches. Cells are colored based on states (left), pseudotime (middle), and PLK1 expression (right). **(E)**. Single-cell trajectory analysis of M1 macrophages reveals seven branches. Cells are colored based on states (left), pseudotime (middle), and PLK1 expression (right).

To further elucidate the correlation between PLK1 and infiltration of immune cells, we performed a single-cell RNA-seq analysis to reveal the expression level of PLK1 in different cell clusters in the tumor microenvironment of gliomas. First, we employed the R package “Seurat” for the processing of the single-cell data expression matrix. In this way, seven cell clusters were annotated, including myeloid cells, neoplastic cells, oligodendrocyte precursor cells, oligodendrocytes, astrocytes, neurons, and vascular cells (**Figures 8A, B**). Then, the expression level of PLK1 was visualized in all clusters. We found that PLK1 was mainly expressed in myeloid cells and neoplastic cells. So, the myeloid cells were further annotated into M1-like and M2-like macrophages (**Figure 8C**). Subsequently, we performed the pseudotime trajectory analysis of neoplastic cells and M1-like macrophages using the “Monocle” R package. In neoplastic cells, four main branches and three branch points were identified, and cells were divided into seven states (**Figure 8D**). In M1-like macrophages, seven main branches and six branch points were identified, and cells were divided into 13 states. Then we observed relatively high PLK1 expression in state one and state three of neoplastic cells (**Figure 8E**). Additionally, the expression level of PLK1 was upregulated in state five and state six of M1 macrophages. Overall, PLK1 is expressed at low levels throughout the development of M1 macrophages and neoplastic cells. However, according to previous research, the mRNA and protein level of PLK1 in glioma tissues is up-regulated. Therefore, PLK1 might be highly expressed in other cells in the tumor microenvironment, implicating the role of the local microenvironment in tumorigenesis.

In addition, we also investigated the effects of PLK1 mRNA levels on the TIM of GBM by screening seven metagenes, namely, HCK, IgG, Interferon, lymphocyte-specific kinase (LCK), MHC-I, MHC-II, and STAT1, which reflect the status of inflammation and immune responses. ***HCK***: This cluster encompasses genes specific for macrophages and cells of the monocyte/myeloid lineage; ***IgG***: Most of the genes in this cluster represent genes of immunoglobulins of the immunoglobulin gamma type mainly associated with B lymphocytes; ***Interferon***: All genes in this cluster represent genes known to be interferon inducible and that are associated with the interferon response of cells; ***LCK***: Genes in this cluster contain T-cell-specific markers; ***MHC-I***: This cluster contains HLA-A, HLA-B, HLA-C, HLA-F and HLA-G genes of the major histocompatability class I complex common to all cell types for the presentation of intracellular antigens; ***MHC-II***: This cluster contains the HLA-DP, HLA-DQ, HLA-DR genes of the major histocompatability class II complex expressed on the surface of professional antigen-presenting cells for their interaction with T cells; ***STAT1***: The genes in this cluster are associated with interferon signal transduction and are also interferon inducible (45). Our results showed that PLK1 mRNA levels were negatively correlated with enrichment scores of Interferon (All genes in this cluster represent genes known to be interferon inducible and that are associated with the interferon response of cells) and LCK (Genes in this cluster contain T-cell-specific markers) (**Supplementary Figure 9**). This suggested that PLK1 might regulate interferon signaling and T cell signaling in GBM.

Overall, our results suggested that PLK1 could regulate immune cells infiltration to glioma TIM and might be a potential immune biomarker of glioma.

### 3.7 Experimental verification of PLK1 expression and phenotype in glioma

Although the results of a series of bioinformatics analyses had confirmed that PLK1 played oncogenic roles in pan-cancer, the experimental verification was more convincing. Therefore, we demonstrated the expression differences and biological roles of PLK1 in normal human astrocyte (HA) cell lines and several glioma cells lines through some experiments, taking glioma as the representative. Moreover, we confirmed the high expression of PLK1 in glioma tissues by RNA sequencing of 100 glioma tissues.

**Figure 9:**
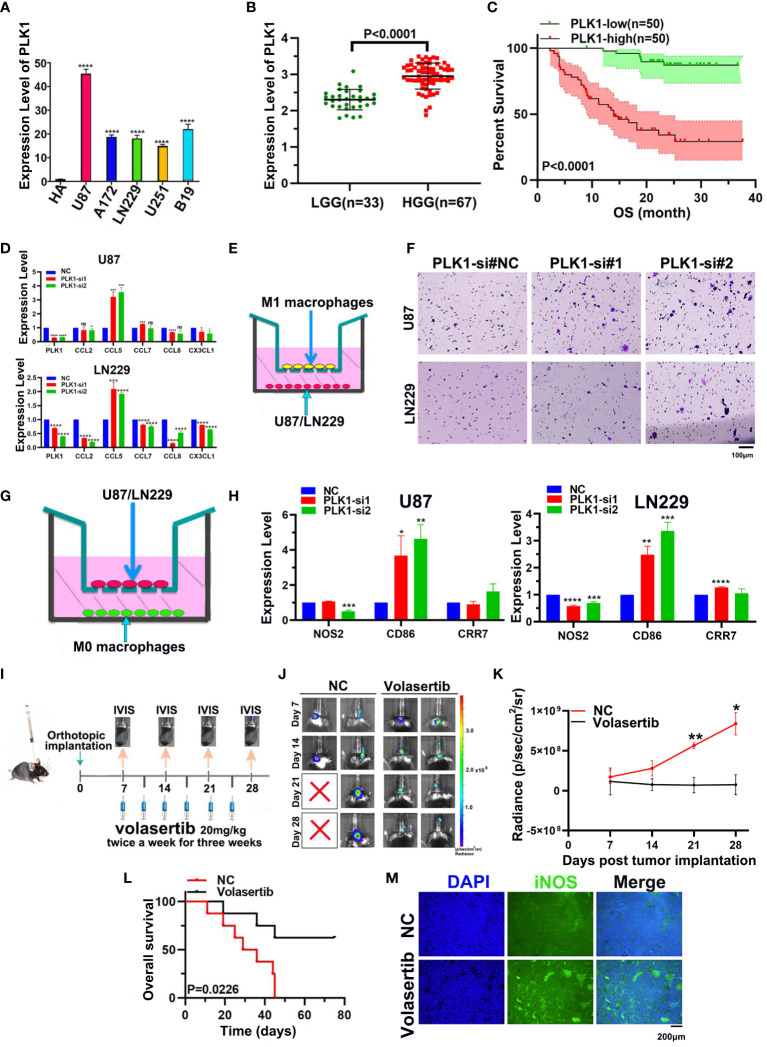
Experimental verification of PLK1 expression and phenotype in glioma. **(A)**. The expression levels of the PLK1 mRNA in NHA cell and five different glioma cell lines (U87, U251, LN229, A172 and B19) by qRT-PCR. **(B)**. The correlation between PLK1 expression and grades in 100 glioma samples, including LGG (n=33) and HGG (n=67) samples; **(C)**. Overall survival (OS) of different PLK1 expression level in 100 glioma samples. **(D)**. The mRNA expression levels of several chemokines of M1 macrophages were detected after knock-down PLK1 in U87 and LN229 cells. **(E)**. The diagram of M1 macrophages infiltration assays. **(F)**. Infiltration of M1 macrophages in si-NC (left), si-PLK1#1 (middle), si-PLK1#2 (right) of U87 and LN229 cells. **(G)**. The diagram of co-culture assays of macrophages and glioma cell lines. **(H)**. The mRNA expression levels of M1 macrophages markers were detected in M0 macrophages derived from THP-1 cells. **(I)**. The scheme of tumor inoculation and systemic injection. **(J)**. Representative tumor bioluminescence images of mice at 7, 14, 21, and 28 days after tumor implantation. **(K)**. Tumor growth curves for mice by quantification of bioluminescent imaging signal intensities. **(L)**. Kaplan-Meier survival analysis of mice bearing orthotopically transplanted 261 cells. **(M)**. Representative image of immunofluorescence (iNOS) in control and volasertib-treated groups. *P<0.05, **P<0.01, ***P<0.001, ****P<0.000.1.

The qRT-PCR showed that PLK1 RNA expression levels in glioma cell lines were significantly higher than that in NHA cells, and the highest expression levels were found in U87 cells (**Figure 9A**). Moreover, analysis of 100 glioma cases collected by our group also showed that PLK1 mRNA level was positively correlated with glioma grade and poorer prognosis, which was consistent with our analysis results in CGGA and TCGA databases (**Figures 9B, C**).

Subsequently, we selected the two cell lines (U87 and LN229) to conduct experiments related to cell proliferation in order to verify the results of enrichment analyses. The PLK1 gene in two glioma cell lines was knocked down with siRNA. Its expression was then confirmed across qRT-PCR (**Supplementary Figure 10**). The EdU assays showed that knockdown of PLK1 led to significantly decreased cell proliferation (**Supplementary Figures 11A, B**). The flow cytometry analyses showed that the percentage of cells in G2/M phase was increased in si-PLK1 group compared to the control group (**Supplementary Figures 11C, D**). These results suggested that si-PLK1 could induce G2/M arrest.

Furthermore, our qRT-PCR showed that CCL5, M1 macrophages chemokines, were significantly overexpressed after downregulating PLK1 in both U87 and LN229 (**Figure 9D**). Consistently, M1 macrophages infiltration was increased after knockdown of PLK1 (**Figures 9E, F**). THP-1 cells were differentiated into M0 and M1 macrophages on the basis of classical inducing methods (**Supplementary Figure 12**). To explore the effect of PLK1 on the polarization of macrophages, we co-cultured the U87/LN229 cells (transfected si-PLK1) with M0 macrophages. The qRT-PCR test confirmed that the expression of M1 macrophages marker CD86 in M0 macrophages was significantly up-regulated in the co-cultured with PLK1 knock-down U87/LN229 cells compared with the control cells. Therefore, these results demonstrated that PLK1 might promote glioma progression by inhibiting M1 macrophages infiltration and polarization (**Figures 9G, H**).

In order to investigate the effect of PLK1 on the TIM of glioma, we established mouse intracranial orthotopic implantation tumor model. Volasertib, a PLK1 inhibitor, can significantly inhibit tumor growth and prolong the survival of animals in intracranial xenograft models (**Figures 9I–L** and **Supplementary Figure 11E**). Moreover, compared with the control group, volasertib can increase the signal of M1 macrophages marker iNOS in the tumor region, which suggested that PLK1 inhibition could increase the M1 macrophages infiltration to glioma TIM (**Figure 9M**).

In conclusion, we verify the conclusions of the above bioinformatics analysis through experiments. That is, PLK1 promotes the malignant characteristics and progression of glioma by accelerating cell proliferation and inhibiting M1 macrophages infiltration and polarization.

### 3.8 The DNA methylation levels of PLK1 in different human cancers

In order to study the mechanism of abnormal expression of PLK1, we also performed PLK1 DNA methylation analysis. DNA methylation of oncogenes usually enhances their expression level and leads to tumor development (46).

The online tool UALCAN was used to explore the methylation level in the PLK1 promoter region. Similar to the above results, PLK1 promoter methylation levels were lower in thyroid carcinoma (THCA), uterine corpus endometrial carcinoma (UCEC), lung adenocarcinoma (LUAD), rectum adenocarcinoma (READ), bladder urothelial carcinoma (BLCA), liver hepatocellular carcinoma (LIHC), esophageal carcinoma (ESCA) and testicular germ cell tumors (TGCT) compared to the normal tissues (**Supplementary Figure 13**). These results may imply that the promoter methylation of PLK1 might contribute its abnormal expression.

Through the analysis of online tool MEXPRESS, we observed that the PLK1 mRNA expression levels were negatively related to the PLK1 methylation levels in both LGG and GBM (**Supplementary Figure 14**). The PLK1 mRNA levels were negatively related to the PLK1 methylation levels at probe ID: cg04138181 (r=-0.274, P<0.001) and probe ID: cg04758185 (r=-0.110, P<0.05) in LGG (**Supplementary Figure 14A**). The mRNA expression levels of PLK1 were negatively related with the methylation levels of PLK1 at probe ID: cg05657488 (r=-0.373, P<0.01) and probe ID: cg04138181 in GBM (r=-0.267, P<0.05) (**Supplementary Figure 14B**). Additionally, the relationship between PLK1 methylation levels and WHO grade of glioma was analyzed across the CGGA database. We also used the CGGA database to analyze the correlation between PLK1 methylation levels and survival of glioma patients. The results indicated that the levels of PLK1 methylation were negatively associated with the WHO grade of glioma. The methylation levels of PLK1 in WHO I gliomas were significantly higher than that in WHO II and WHO IV gliomas (**Supplementary Figure 14C**). In the survival analysis of primary glioma samples, the lower levels of PLK1 methylation were associated with poorer prognosis (**Supplementary Figure 14D**).

In summary, these results suggested that low methylation levels of PLK1 might contribute to its overexpression in pan-cancer, especially in glioma.

### 3.9 Construction of the upstream lncRNA-miRNA regulatory network that regulates PLK1 expression in glioma and other tumors

In recent years, several studies have shown that long non-coding RNAs (lncRNAs) play a significant role in tumorigenesis by regulating the expression of the downstream mRNAs through sequestering of their target miRNAs. Therefore, we investigated the lncRNA-miRNA network that may regulate PLK1 expression in various tumors especially in glioma. First, we screened the miRWalk, TargetScan, and miRmap databases and identified 47 miRNAs that potentially target the PLK1 mRNAs (**Supplementary Figure 15A**). The top 10 PLK1 mRNA-targeting miRNAs were hsa-miR-296-5p, hsa-miR-92a-2-5p, hsa-miR-3665, hsa-miR-4660, hsa-miR-1185-1-3p, hsa-miR-1185-2-3p, hsa-miR-509-3-5p, hsa-miR-509-5p, hsa-miR-3120-3p and hsa-miR-4728-5p (**Supplementary Figure 15B**).

Among these 10 PLK1 mRNA-targeting miRNAs, 4 miRNAs (hsa-miR-296-5p, hsa-miR-92a-2-5p, hsa-miR-509-3-5p, and hsa-miR-509-5p) were found in the CGGA database. Then, we analyzed the relationship between these 4 miRNAs expression levels and prognosis and glioma grades in CGGA dataset (**Supplementary Figures 15C, D, 16**). The results showed that hsa-miR-296-5p and hsa-miR-92a-2-5p expression levels had statistic relationship with both prognosis and grades. Moreover, hsa-miR-92a-2-5p expression level associated with better prognosis and the hsa-miR-92a-2-5p expression level was negatively associated with glioma grades, which implied that hsa-miR-92a-2-5p was tumor suppressor in glioma. Therefore, these results suggested that hsa-miR-92a-2-5p potentially targeted PLK1 mRNA in glioma.

Next, we identified lncRNAs that may target hsa-miR-92a-2-5p using the TargetScan database. The top 10 predicted lncRNAs and top 10 validated lncRNAs were used to construct a lncRNA-miRNA-PLK1 regulatory network using the cytoscape software (**Supplementary Figure 15E**). These results demonstrated the upstream lncRNA-miRNA regulatory network that may regulate the aberrant expression of PLK1 in the glioma.

## 4 Discussion

PLKs, in mammals, has diverged into five paralogues, PLK1-5. It has been widely reported that PLK1 is a member of PLK kinases family and plays important roles in cell cycle and mitosis (47). A large number of newly-presented literatures have reported that aberrant expression of PLK1 may lead to many clinical diseases, especially cancers (48). Whether PLK1 can exert effects in the pathological process of various cancers through some similar molecular mechanisms has yet to be explored. By searching literatures, we found that there was little analysis of the oncogenic roles of PLK1 in pan-cancer. Therefore, we used a number of databases to detect the molecular features of PLK1 gene expression, gene alteration, immune infiltration, cell cycle in pan-cancer, especially glioma. It is worth noting that PLK1 affects the TIM of glioma by regulating M1 macrophages infiltration. What’s more, we also explored the potential molecular mechanisms of PLK1 aberrant expression by analyzing methylation of PLK1 DNA and ceRNA network in glioma.

Firstly, we explored the expressions of PLK1 in various cancers and normal tissues since many previous studies have claimed that abnormal expressions of PLK1 can attract the occurrence of numerous diseases, including cancers (49). PLK1 was highly expressed in both mRNA and protein levels in several tumors (**Figures 1**, **2**). The pan-cancer analysis also showed statistic correlation between PLK1 expression level and the prognosis (**Figure 3**). Since our specialty was neuro-oncology, we subsequently conducted a detailed study on the relationship between PLK1 expression and prognosis in glioma. In our studies, high PLK1 level was also associated with clinical features such as grade, IDH mutation status, 1p/19q co-deleted status, and methylation status of MGMT promoter in glioma (**Supplementary Figure 2**). RNA-seq of 100 glioma cases in our sample database discovered that glioma patients with high PLK1 level had poorer prognosis compared with patients with low PLK1 level (**Figure 9C**), which was consistent with the results using CGGA and TCGA datasets (**Supplementary Figure 3A**). Cox regression analysis showed that PLK1 was an independent prognostic predictor in glioma (**Supplementary Figure 4**). Recent research reported that aberrant upregulated PLK1 correlates with recurrence and poor prognosis in colorectal cancer patients (50). Qian et al. validated that PLK1 was highly expressed in clear cell renal cell carcinoma (ccRCC) tissues and promoted ccRCC cell proliferation, migration, invasion, and cell cycle (51). He et al. reported that PLK1 was highly expressed and predicted a poor prognosis in hepatocellular carcinoma patients (52). Similarly, Wang et al. demonstrated that PLK1 levels were elevated in glioma compared with those in normal brain tissues, and high expression of PLK1 was associated with poor prognosis (53). Overall, our analysis showed that PLK1 was highly expressed and a potential prognostic biomarker in various cancers, especially in glioma.

Secondly, in order to explore the oncogenic mechanisms of PLK1 in pan-cancer, the top 100 PLK1 positively correlated genes in pan-cancer were obtained to perform enrichment analysis (**Figure 4**). Consistent with the results in pan-cancer, PLK1-related genes were enriched not only in classical carcinogenic signaling pathways and terms but also in cell cycle, genetic alteration and immune related pathways and terms in glioma (**Supplementary Figures 6, 7**). Therefore, we mainly explored the molecular mechanism of PLK1 exerting oncogenic effects by regulating cell cycle, genetic alteration and immune microenvironment in detail.

*PLK1* gene alteration was the most frequent in UCEC patients among 32 cancer types, and was mainly represented as mutation. Notably, missense mutation was the most abundant form of *PLK1* gene alteration, and it mainly manifested as the mutation at the site 293 of PLK1 protein from R(Arginine) to C(Cysteine)/H(Histidine) (**Supplementary Figures 8A–C**). Gao et al. reported that the predominant type of mutation for PLK1 was missense mutation in cervical cancer, which consistent with the result of our pan-cancer analysis. In addition, since the R293C/H missense mutation occurred in the Pkinase domain of PLK1 protein, we speculated that the R293C/H missense mutation could lead to the genesis and progression of cancers by changing the Pkinase activity of PLK1 proteins. Nevertheless, we have not found the R293C/H missense mutations of PLK1 in previous studies. Therefore, the R293C/H missense mutation of PLK1 may serve as a new potential direction for tumor research in the future. Next, we explored the correlation between gene alterations of characteristic molecules and PLK1 expression level in glioma. Isocitrate dehydrogenase (IDH) mutation and chromosomal 1p/19q codeletions are associated with better survival outcomes of glioma patients. The chromosomes 7/10 were also molecular profiles characteristically altered in glioblastoma multiforme (GBM) according to the 2021 WHO classification of CNS tumors (54). In brief, amplification of chromosome 7 and deletion of chromosome 10 predicts poor prognosis in patients with glioma. The co-deletion of 1p/19q was significant, and the IDH mutation rate was up to 75% in glioma with PLK1 low expression. The amplification of chromosome 7 and deletion of chromosome 10 were significant in glioma with PLK1 high expression with high mutation rate of TP53 (38%) (**Supplementary Figures 8D–H**). These results suggest that both the variation of PLK1 itself and other gene variations related to PLK1 expression have an impact on the occurrence and progression of tumors, especially glioma.

Following, increasing literatures showed that immune effects played critical roles in anti-tumor mechanisms and may serve as new diagnostic and therapeutic potential targets in cancers. Several studies have shown that tumor-infiltrating immune cell/tumor-infiltrating lymphocytes (TIIs/TILs) are important components of the tumor microenvironment. Moreover, immunity/inflammatory-related metagenes can help tumor to obtain multiple hallmarks and regulate the tumor progression. Therefore, the association between PLK1 gene expression levels and tumor microenvironment was analyzed. The results of pan-cancer analysis implied that PLK1 expression levels were negatively corelated with the multiple TIIs/TILs levels in several cancer types (**Figures 5B, C**). Similarly, Takeshita et al. found that PLK1 mRNA expression was significantly associated with CD8+T cells, activated memory CD4+T cells, M0 macrophage, M1 macrophage, and M2 macrophage in ER positive Her2 negative breast cancer (55). And Park et al. claimed that PLK1 could be a universal tumor antigen recognized by cytotoxic T lymphocytes for cancer immunotherapy in murine tumor models (56). ESTIMATE analysis showed that the expression of PLK1 negatively correlated with the infiltration of immune cells and stromal cells in glioblastoma multiforme (GBM) (**Figure 6**). Similarly, PLK1 expression levels were negatively corelated with infiltration levels of multiple immune cells, especially with M1 macrophages in GBM in TCGA datasets (**Figures 7A, B**). These results suggested that PLK1 inhibited the infiltration of immune cells into glioblastoma multiforme (GBM), thereby enabling the tumor cells to evade the immune system. Zhou et al. found that inhibiting PLK1 could alter the tumor immune microenvironment by enriching T cells infiltration non-small cell lung cancer (NSCLC), which was consistent with our conclusion (57). In our study, we also found that PLK1 expression was negatively with the expression of M1 macrophage chemokines and the infiltration levels of M1 macrophages (**Figure 7**). What’s more, we verified that knockdown of PLK1 could increase the expression of the M1 macrophage chemokine CCL5 in glioma cell lines U87 and LN229 (**Figure 9D**). Interestingly, PLK1-knockdown U87/LN229 cells promoted the migration and polarization of M1 macrophages (**Figures 9E-H**). And PLK1 inhibitor volasertib can promote M1 macrophages infiltration to tumor region *in vivo* (**Figure 9M**). Therefore, we hypothesized that PLK1 might inhibit M1 macrophages polarization and infiltration, which the specific molecular mechanism is still being explored.

Our results about immunity/inflammatory-related metagenes showed that PLK1 mRNA levels were negatively correlated with enrichment scores of Interferon and lymphocyte-specific kinase (LCK) in glioblastoma multiforme (GBM) (**Supplementary Figure 9**), which implied that Interferon and LCK might be protective factors, different from PLK1. A previous study reported that “Immunity” metagene was associated with a better prognosis in HER2-positive/ER-negative breast cancers, which was consistent with our results (58). Callari et al. claimed that the IFN metagene was associated with a low risk of metastasis in 104 ERBB2+ tumors (59). Similarly, Ma et al. confirmed that the survival rate of patients with high LCK metagene expression was markedly higher than that of the low expression group in the endometrioid endometrial adenocarcinoma subtype group (60).

Furthermore, qRT-PCR has verified that PLK1 is high expressed in glioma cell lines and tissues (**Figures 9A, B**). And flow cytometry and EdU assays have verified that PLK1 can accelerate cell cycle and stimulate cell proliferation in glioma cell lines (**Supplementary Figures 11A–D**). Similarly, Wu et al. confirmed that of PLK1 significantly promoted cell proliferation, migration, invasion, and inhibited apoptosis of U87 and U251 glioma cells (61). Cheng et al. reported that down-regulation of PLK1 could inhibit growth, induce cell arrest in G2/M phase of cell cycle and apoptosis enhancement in glioma cells (62). Interestingly, some studies have shown that PLK1 inhibitors could inhibit glioma cell proliferation and glioma progression. Such as, Li et al. reported that BI2536 (PLK1 inhibitor) could diminish glioma stem cells (GSC) self-renewal *in vitro*, and increase survival of orthotopic tumor-bearing mice (63). Similarly, PLK1 inhibitor volasertib significantly inhibited tumor proliferation, prolonged the survival of animals, and increased the infiltration of M1 macrophages in tumor region in our mouse model of intracranial xenograft tumors (**Figures 9I–M**).

Lastly, to explore possible molecular mechanisms of aberrant expressions of the PLK1, we attempted to analyze whether PLK1 DNA methylation and ceRNA network could affect PLK1 expression levels. It has been reported that gene methylation often leads to low expression levels. Compared with normal tissue, the PLK1 gene promoter region was less methylation in several tumors (**Supplementary Figure 13**). Taking glioma as an example, the methylation level of PLK1 was negatively correlated with PLK1 expression level (**Supplementary Figures 14A, B**). Furthermore, high methylation levels of PLK1 contributed to poor prognosis and advanced grades in glioma patients (**Supplementary Figures 14C, D**). Consistent with our analysis, a previous study reported PLK1 which was typically hypermethylated in normal liver tissue but became hypomethylated and upregulated in liver tumor (64). In addition, we also conducted a lncRNA-miRNA regulatory network that may regulate the aberrant expression of PLK1 in glioma (**Supplementary Figures 15, 16**). The analysis results suggested that the gene methylation and ceRNA regulatory network of PLK1 might be the important molecular mechanisms that contribute to the aberrant expressions of PLK1.

In summary, our results suggested that PLK1 was overexpressed in various cancers and significantly correlated with the poorer prognosis. The results of bioinformatics analysis indicated that gene alteration and anti-tumor immunity might be the potential oncogenic mechanisms of PLK1 in pan-cancer, especially glioma. *In vivo* and *in vitro* experiments confirmed that PLK1 affected glioma progression and TIM by regulating the infiltration and polarization of M1 macrophages. The analysis results implied that hypo-methylation of PLK1 and abnormal regulation of ceRNA network are responsible for its abnormal expression. In conclusion, our present study suggested that PLK1 may have potential as a diagnosis and prognostic marker as well as immunotherapeutic targets for several malignant tumors, especially glioma.

## Data availability statement

The datasets presented in this study can be found in online repositories. The names of the repository/repositories and accession number(s) can be found in the article/**Supplementary Material**.

## Ethics statement

The studies involving human participants were reviewed and approved by Zhengzhou University. The patients/participants provided their written informed consent to participate in this study. The animal study was reviewed and approved by Zhengzhou University. Written informed consent was obtained from the individual(s) for the publication of any potentially identifiable images or data included in this article. 

## Author contributions

LH, X-ZL, and Z-YZ conceived the review. LL, X-YZ and Y-WZ drafted the manuscript and revised it before submission. X-ZL, D-ZP, and Z-YZ collected the references. LL, LH and X-YZ performed the experiments. Y-WZ, CW, and G-CG contributed reagents/materials/analysis tools. D-LP revised the manuscript. BY and Y-CJ provided the data. All authors contributed to the article and approved the submitted version. 

## Supplementary material

Supplementary Table 1 Clinical data of glioma patients.

Supplementary Table 2 PLK1 expression in cancers verus normal tissue in oncomine database.

Supplementary Table 3 PLK1 positively related genes in pan-cancer in GEPIA2 database.

Supplementary Table 4 The mRNA-seq data (sheet 1) and clinical data (sheet 2) of CGGA-325 dataset.

Supplementary Table 5 The mRNA-seq data (sheet 1) and clinical data (sheet 2) of CGGA-693 dataset.

Supplementary Table 6 The mRNA-seq data (sheet 1) and clinical data (sheet 2) of TCGA dataset.
