# Supplementary material for: Polo-like kinase 1 is related with malignant characteristics and inhibits macrophages infiltration in glioma
Source: Front Immunol. 2022 Dec 21;13:1058036. doi: 10.3389/fimmu.2022.1058036 (PMC9811677; doi:10.3389/fimmu.2022.1058036)
Supplement: Supplementary file 7 [file DataSheet_1.zip › Supplementary Figures/Supplementary Figure Legends.docx]

**Supplementary Figure Legends**

**Supplementary Figure 1.** **The flowchart of this study.**

**Supplementary Figure 2.** **PLK1 expression in different glioma subtypes.**

(A). The relationship between PLK1 expression and WHO grades of gliomas in CGGA_325, CGGA_693 and TCGA databases. (B). Receiver operating characteristic (ROC) curve to assess sensitivity and specificity of PLK1 expression as a diagnostic biomarker for WHO grades of gliomas. (C). The relationship between PLK1 expression and histologic classification of gliomas in CGGA_325, CGGA_693 and TCGA databases. The expression level of PLK1 in different IDH status (D), 1p/19q status (E) and MGMT promotor status (F) of gliomas in CGGA_325, CGGA_693 and TCGA databases.

**Supplementary Figure 3.** **Correlation between PLK1 expression and prognosis of glioma patients.**

The relationship between PLK1 expression and OS of pan-glioma (A), LGG (B), and HGG (C) in CGGA_325, CGGA_693 and TCGA databases. (D). Receiver operating characteristic (ROC) curve to assess sensitivity and specificity of PLK1 expression as a prognostic biomarker for pan-glioma. (E). The relationship between PLK1 expression and DSS of pan-glioma, LGG, and HGG in TCGA databases. (F). The relationship between PLK1 expression and PFI of pan-glioma, LGG, and HGG in TCGA databases.

**Supplementary Figure 4.** **Univariate and multivariate Cox analyses of PLK1 and clinical features in glioma.**

(A). Multivariate Cox proportional-hazards model survival analysis of various parameters in CGGA_325, CGGA_693 and TCGA databases. (B). The Nomogram model of PLK1 and clinical features in glioma in CGGA_325, CGGA_693 and TCGA databases.

**Supplementary Figure 5.** **The relationships between PLK1 expression and prognosis of glioma patients.**

Kaplan-Meier analysis of OS based PLK1 expression in different prognostic factors including IDH (A), 1p19q (B), MGMT (C), radiotherapy (D), and chemotherapy (E) in CGGA and TCGA datasets.

**Supplementary Figure 6. Enrichment analysis of the PLK1-correlated genes in CGGA, TCGA and FAHZZU datasets.**

The heat map and enrichment analysis of the genes correlated with PLK1 in glioma in CGGA-325 (A), TCGA (B) and FAHZZU (The First Affiliated Hospital of Zhengzhou University) datasets.

**Supplementary Figure 7.** **Enrichment analysis of the PLK1-correlated genes in GEO datasets.**

The volcano figure and enrichment analysis of the genes correlated with PLK1 in glioma in GSE67102 (A) and GSE46856 (B) datasets.

**Supplementary Figure 8. The DNA alterations of PLK1 in pan-cancer.**

The alteration frequency with mutation type (A) and mutation site (B) are displayed using the cBioPortal tool in pan-cancer. The mutation site with the highest alteration frequency in the 3D structure of PLK1 is presented across the cBioPortal tool in pan-cancer (C). (D). Overall copy number variation (CNV) profile according to high vs low PLK1 expression. Blue (deletion); red (amplification). (E). Frequency of specific changes based on PLK1-low (left row) and PLK1-high (right row) groups. The Y-axis represents the frequency of chromosomal amplification (red). (F). Frequency of specific changes based on PLK1-low (left row) and PLK1-high (right row) groups. The Y-axis represents the frequency of chromosomal deletion (blue). (G). Spectrum of somatic mutations in gliomas from PLK1-low groups. (H). Spectrum of somatic mutations in gliomas from PLK1-high groups.

**Supplementary Figure 9.** **Relationship between PLK1 expression and** **inflammatory-related metagenes in glioma.**

Enrichment scores of seven immune and inflammatory-related metagenes in glioma patients with different PLK1 mRNA expression level status in CGGA-325 (A), CGGA-693 (B) and TCGA (C) datasets. The red dots represent the positive correlation between PLK1 and metagenes, while the blue dots represent the negative correlation between PLK1 and metagenes. **P*<0.05, ***P*<0.01, ****P*<0.001, *****P*<0.0001.

**Supplementary Figure 10. Verification of knockdown efficiency effects of PLK1 in U87 and LN229 cell lines.**

The knockdown efficiency effects of PLK1 in U87 and LN229 cell lines. *****P*<0.0001.

**Supplementary Figure 11. The biological functions of PLK1 in glioma.**

(A-B). EdU assays were employed to measure cell proliferation. (C-D). Cell cycle distribution was evaluated using flow cytometry. (E). Representative images of H&E staining for tumor volume in the mouse intracranial orthotopic implantation tumor model for four weeks after tumor implantation. **P*<0.05, ***P*<0.01.

**Supplementary Figure 12. Morphological changes of THP1 cells induced by PMA, LPS and** **IFN-γ.**

Morphological changes of THP1 cells were observed 48 h after PMA induction and 48 h after LPS and IFN-γ induction.

**Supplementary Figure 13.** **The promoter methylation levels of PLK1 in different cancer types compared to normal adjacent tissues.**

**Supplementary Figure 14. DNA methylation levels of PLK1 in glioma.**

PLK1 expression was negatively correlated with PLK1 DNA methylation in LGG (A) and GBM (B) across MEXPRESS. (C). Relationship between PLK1 methylation level and glioma WHO grades in CGGA datasets. (D). Relationship between PLK1 methylation level and prognosis in primary glioma in CGGA database.

**Supplementary Figure 15. The lncRNA-miRNA-PLK1 regulatory network constructed.**

(A). The upstream miRNAs of PLK1 were predicted by miRmap, miRwalk, and TargetScan databases and the intersection was taken (47 intersection miRNAs). (B). The top 10 miRNAs targeting PLK1 were displayed among the 47 miRNAs. (C). The relationship between hsa-miR-92a-2-5p expression and prognosis in glioma patients from CGGA microRNA array dataset. (D) The relationship between hsa-miR-92a-2-5p expression and grades in glioma from CGGA microRNA array dataset. (E). The lncRNA-miRNA-PLK1 regulatory network was constructed by CytoScape.

**Supplementary Figure 16. The relationship between PLK1-related microRNAs and prognosis or grades.**

The relationship between hsa-miR-296-5p (A), hsa-miR-509-3-5p (C), and hsa-miR-509-5p (E) expression and prognosis in glioma from CGGA microRNA array dataset. The relationship between hsa-miR-296-5p (B), hsa-miR-509-3-5p (D), and hsa-miR-509-5p (F) expression and grades in glioma patients from CGGA microRNA array dataset.
