# Supplementary figures and images for: Polo-like kinase 1 is related with malignant characteristics and inhibits macrophages infiltration in glioma
Source: Front Immunol. 2022 Dec 21;13:1058036. doi: 10.3389/fimmu.2022.1058036 (PMC9811677; doi:10.3389/fimmu.2022.1058036)

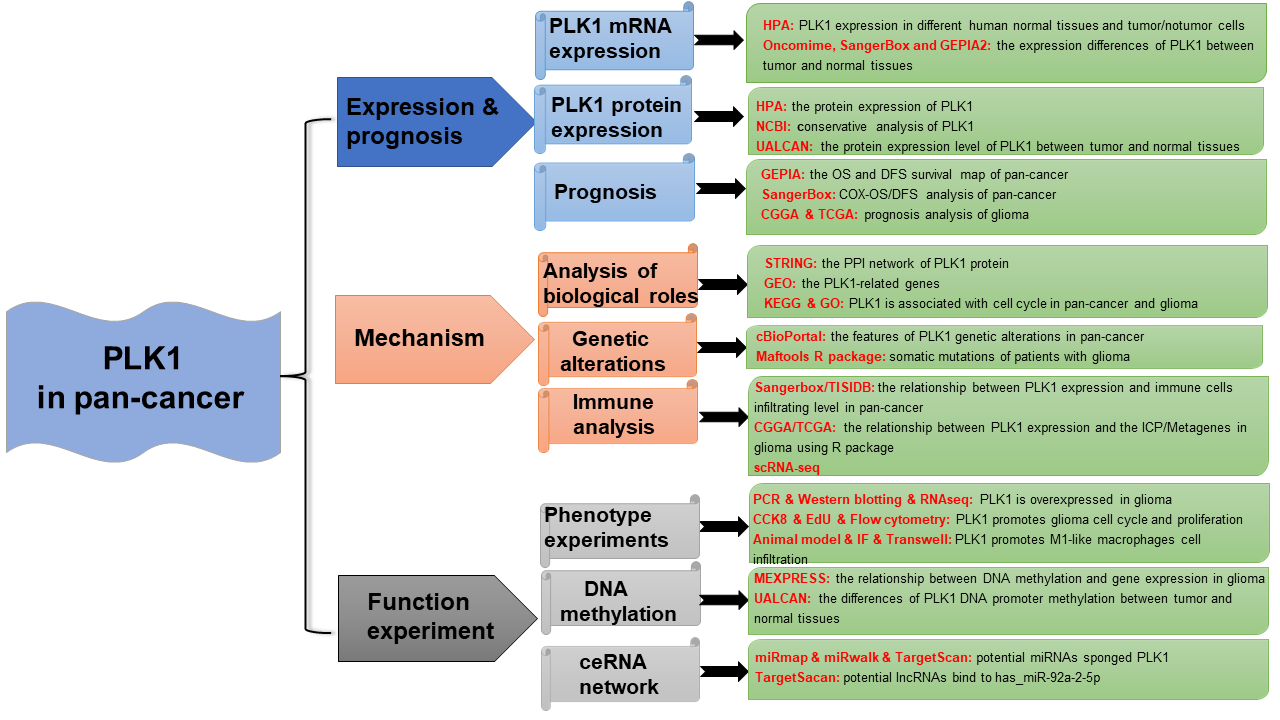

Supplement: Supplementary file 7 [file DataSheet_1.zip › Supplementary Figures/Supplementary Figure 1.tif]

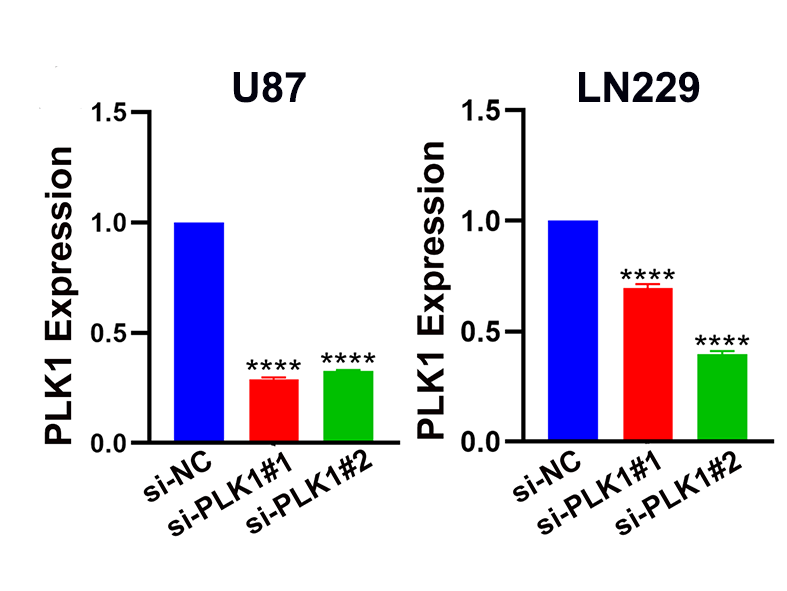

Supplement: Supplementary file 7 [file DataSheet_1.zip › Supplementary Figures/Supplementary Figure 10.tif]

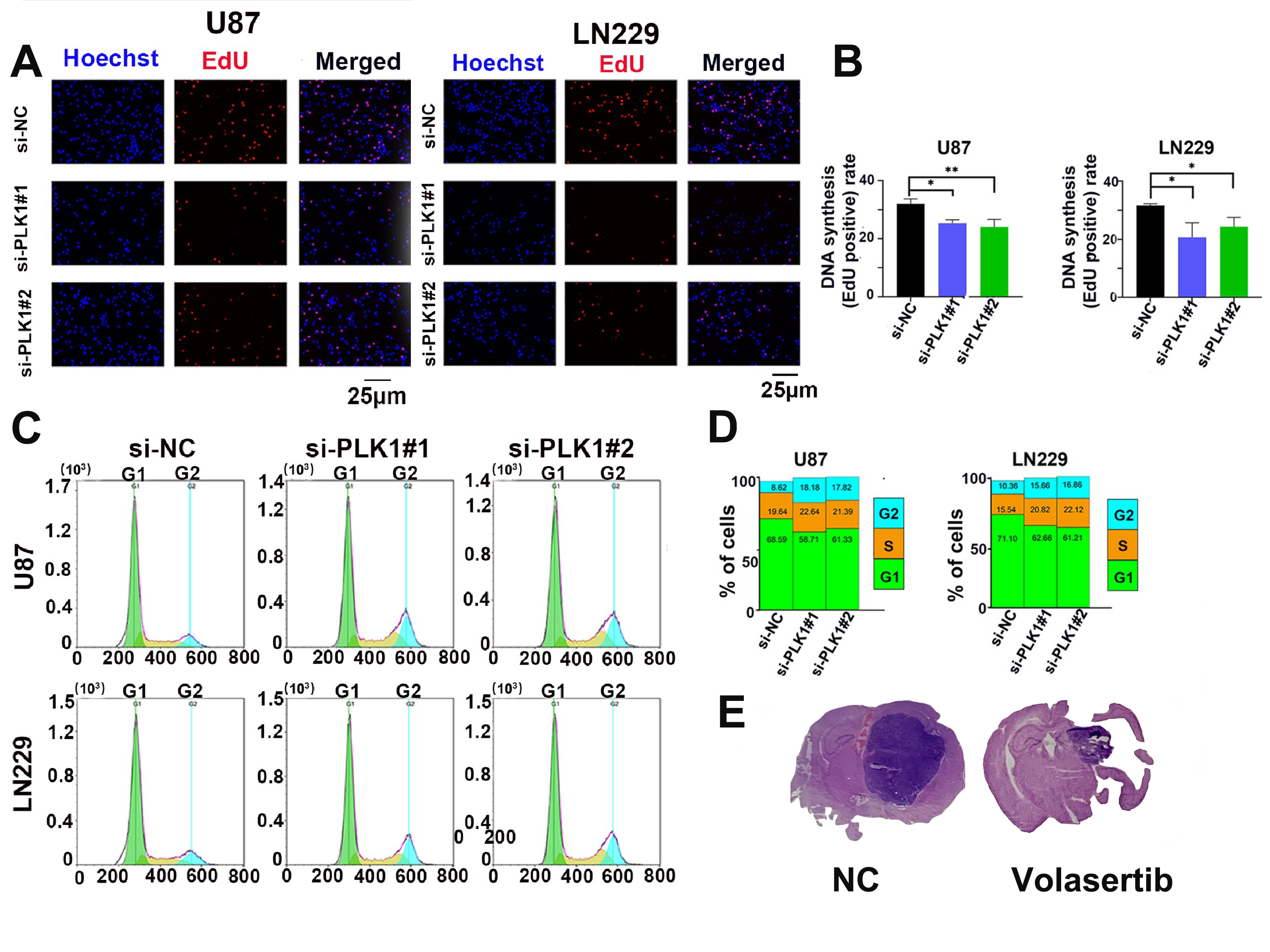

Supplement: Supplementary file 7 [file DataSheet_1.zip › Supplementary Figures/Supplementary Figure 11.tif]

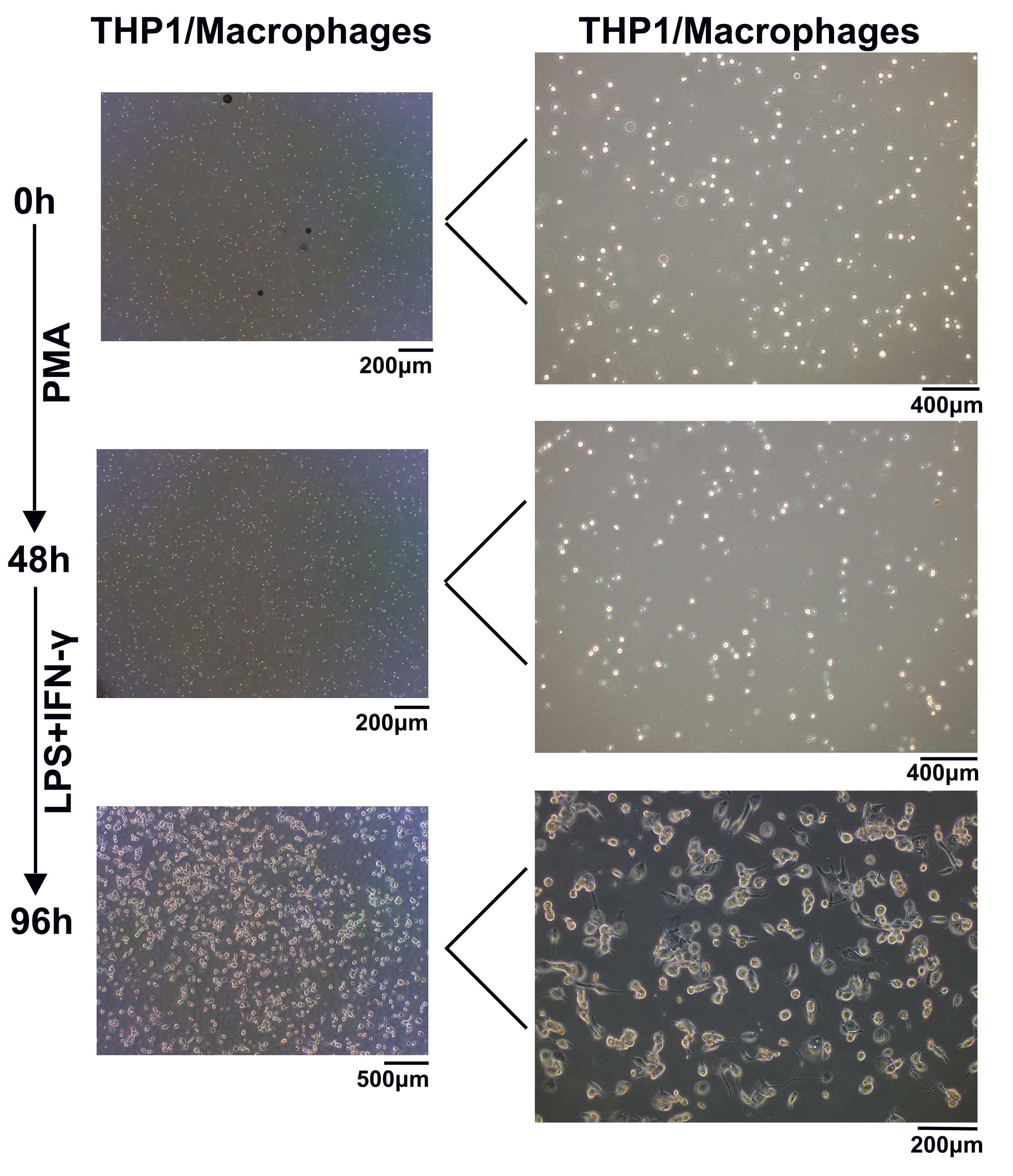

Supplement: Supplementary file 7 [file DataSheet_1.zip › Supplementary Figures/Supplementary Figure 12.tif]

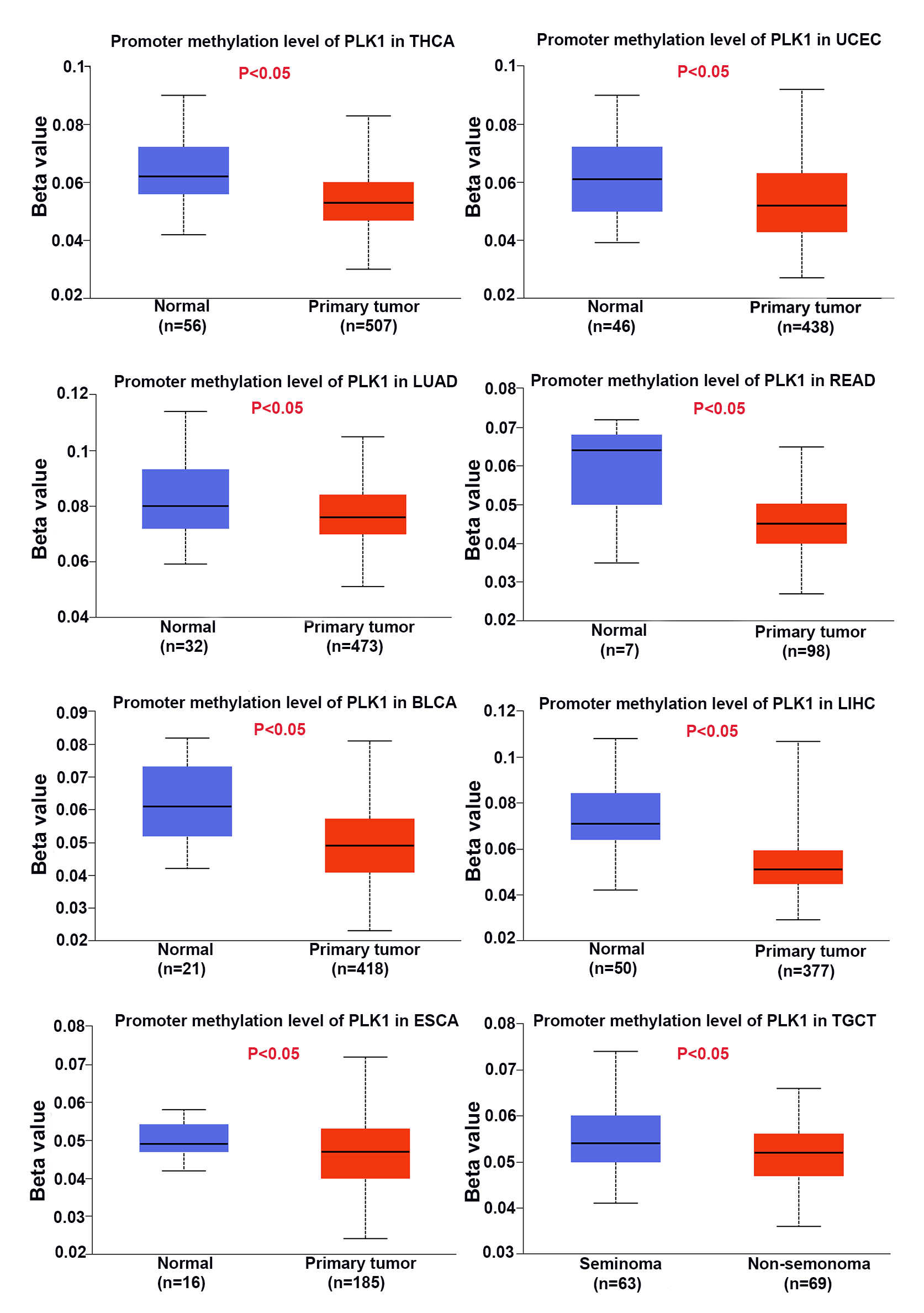

Supplement: Supplementary file 7 [file DataSheet_1.zip › Supplementary Figures/Supplementary Figure 13.tif]

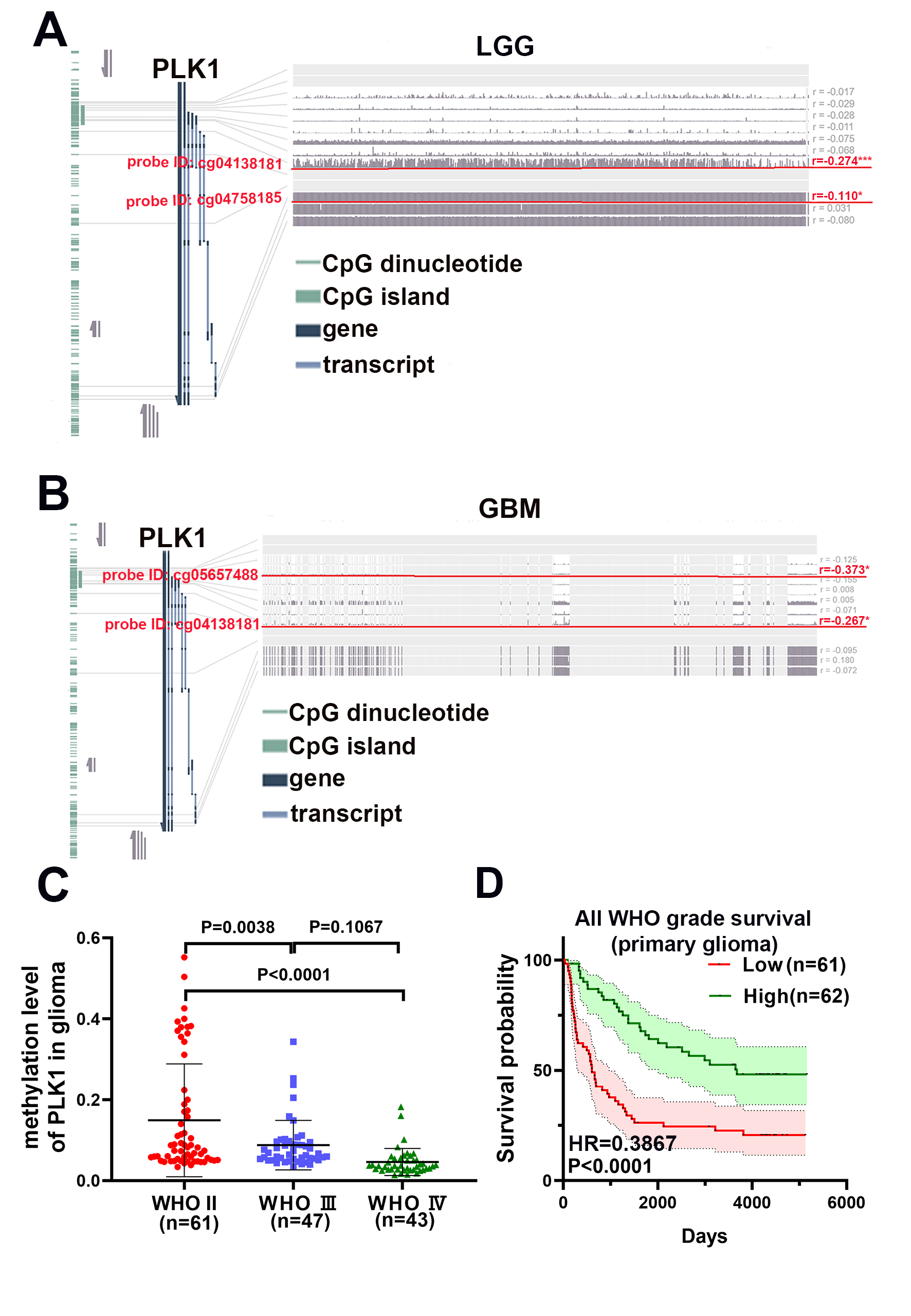

Supplement: Supplementary file 7 [file DataSheet_1.zip › Supplementary Figures/Supplementary Figure 14.tif]

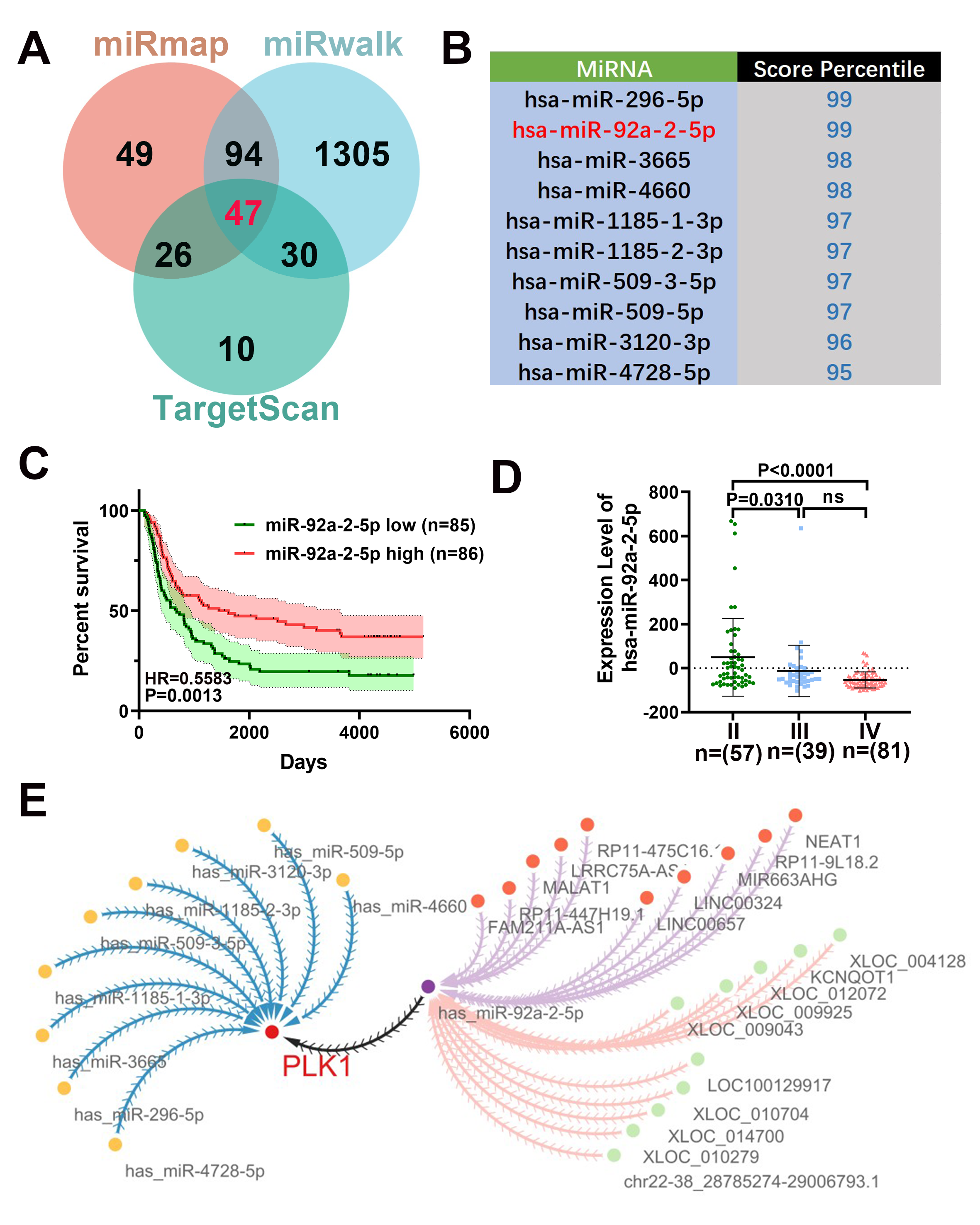

Supplement: Supplementary file 7 [file DataSheet_1.zip › Supplementary Figures/Supplementary Figure 15.tif]

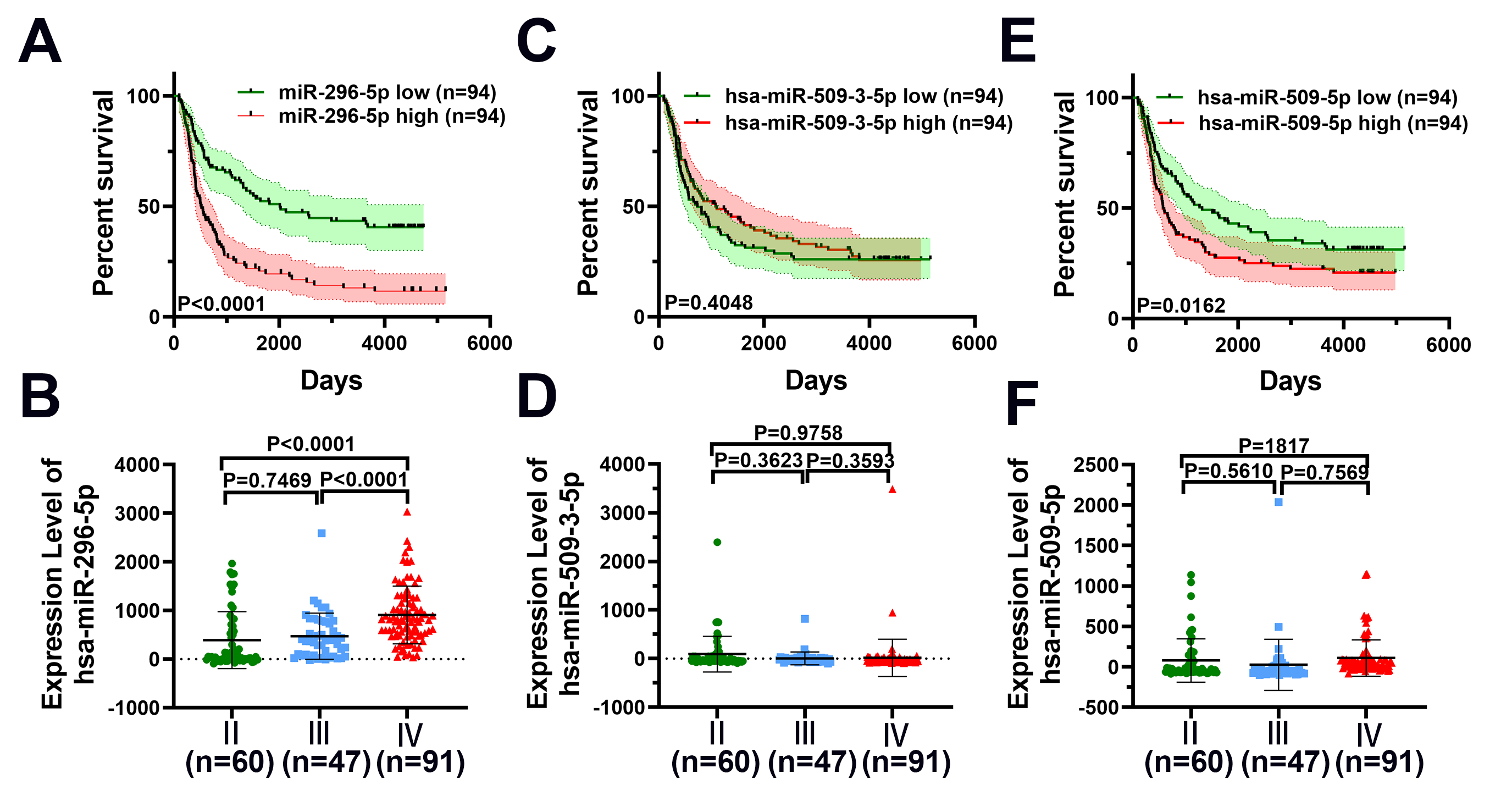

Supplement: Supplementary file 7 [file DataSheet_1.zip › Supplementary Figures/Supplementary Figure 16.tif]

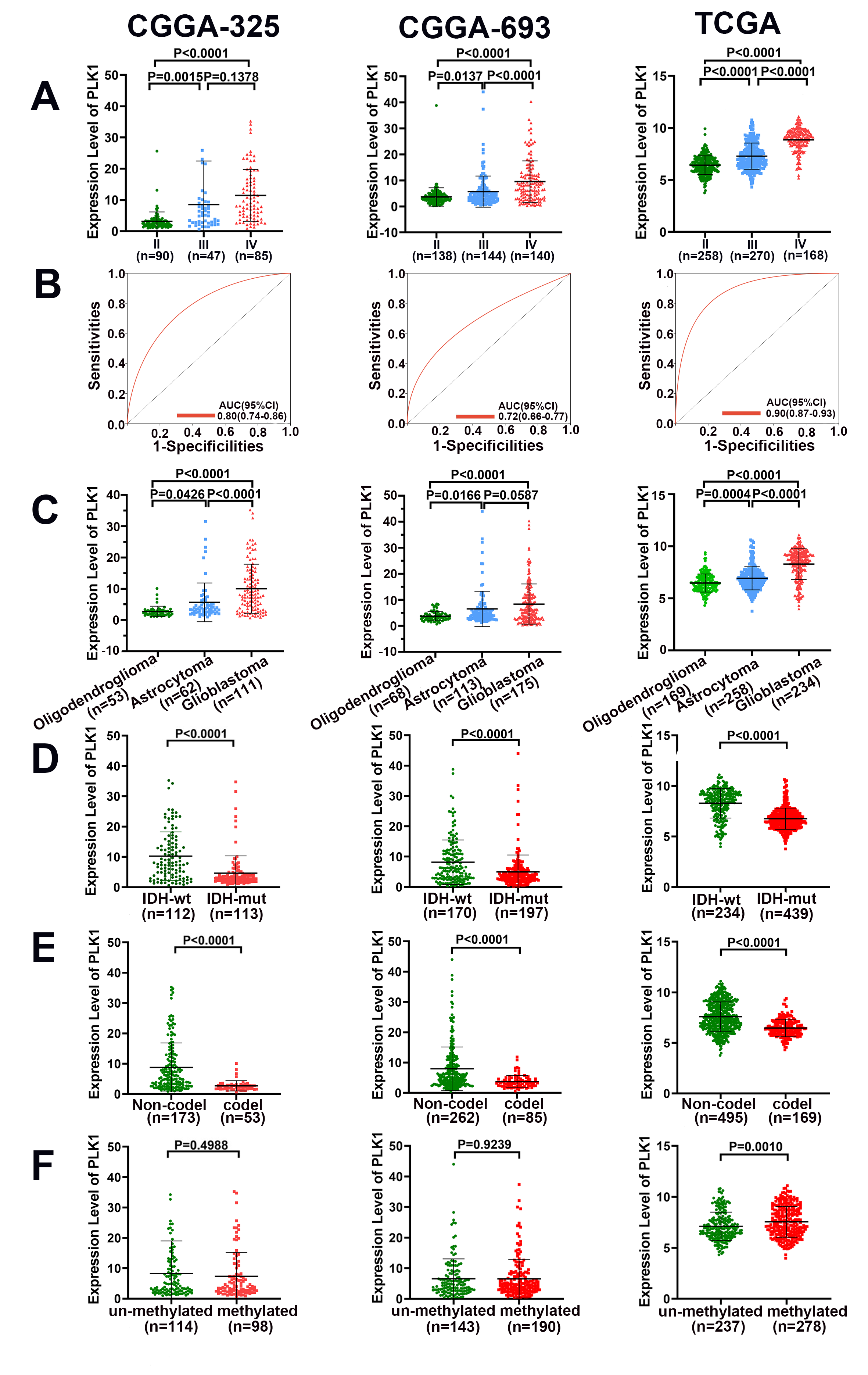

Supplement: Supplementary file 7 [file DataSheet_1.zip › Supplementary Figures/Supplementary Figure 2.tif]

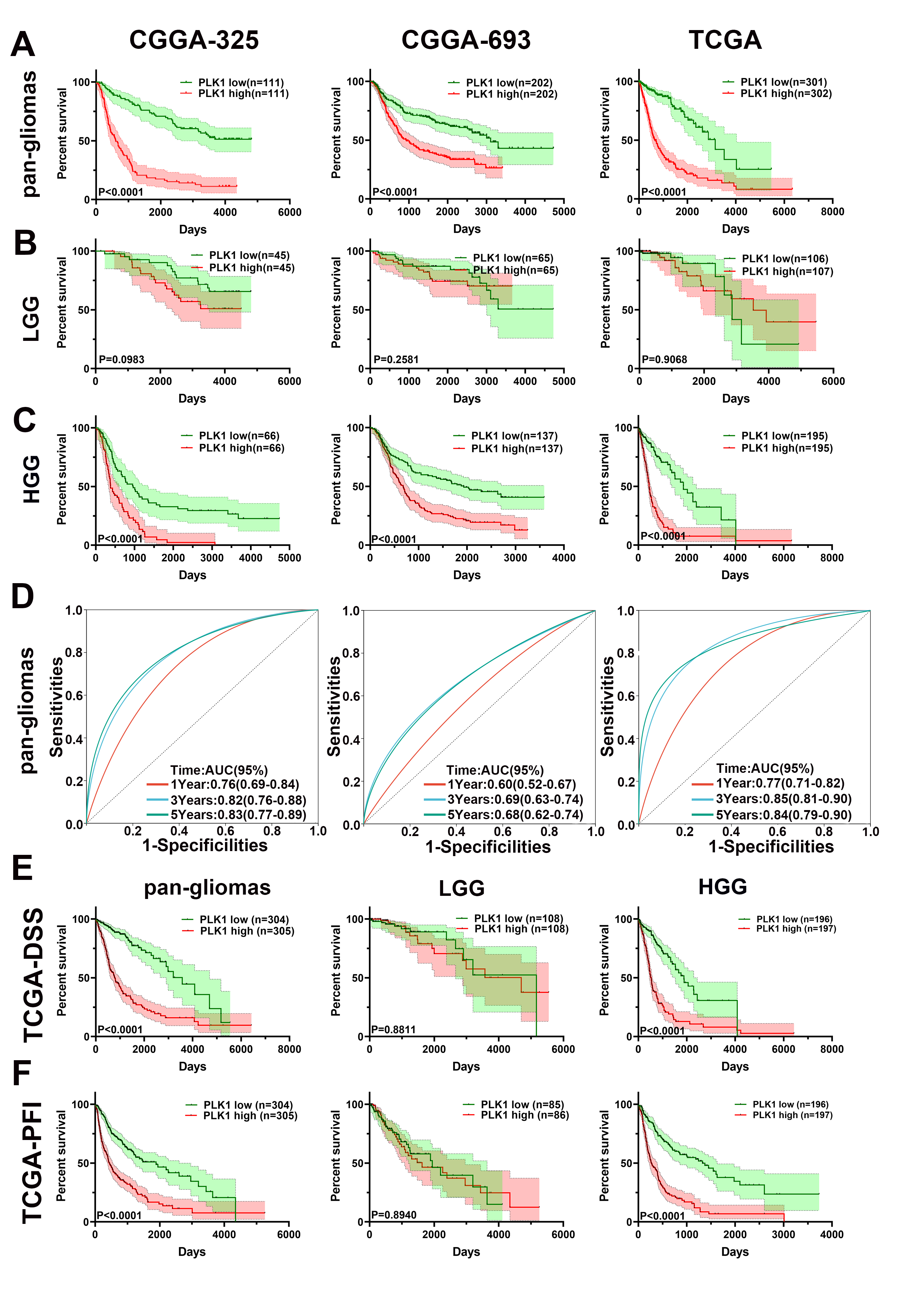

Supplement: Supplementary file 7 [file DataSheet_1.zip › Supplementary Figures/Supplementary Figure 3.tif]

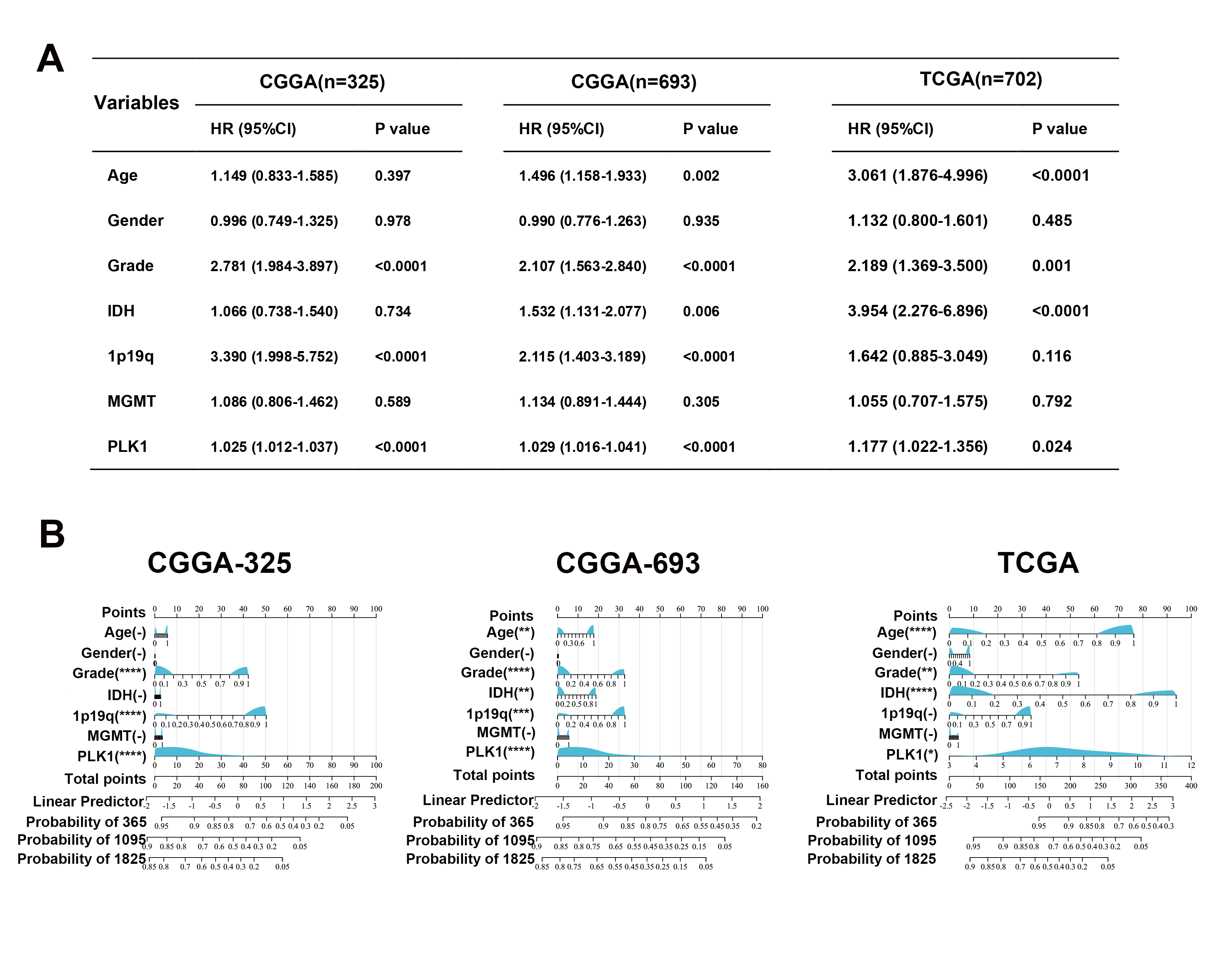

Supplement: Supplementary file 7 [file DataSheet_1.zip › Supplementary Figures/Supplementary Figure 4.tif]

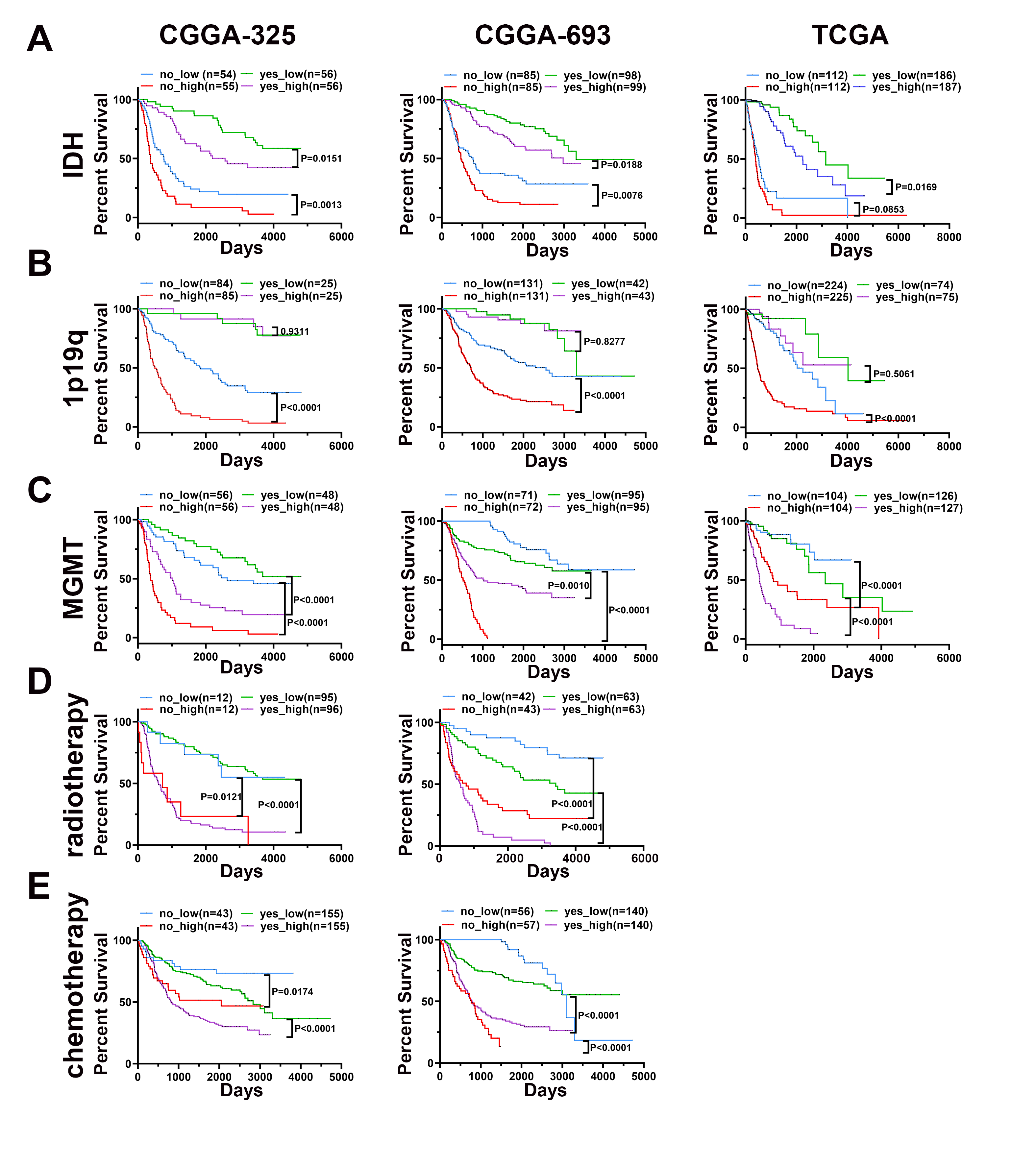

Supplement: Supplementary file 7 [file DataSheet_1.zip › Supplementary Figures/Supplementary Figure 5.tif]

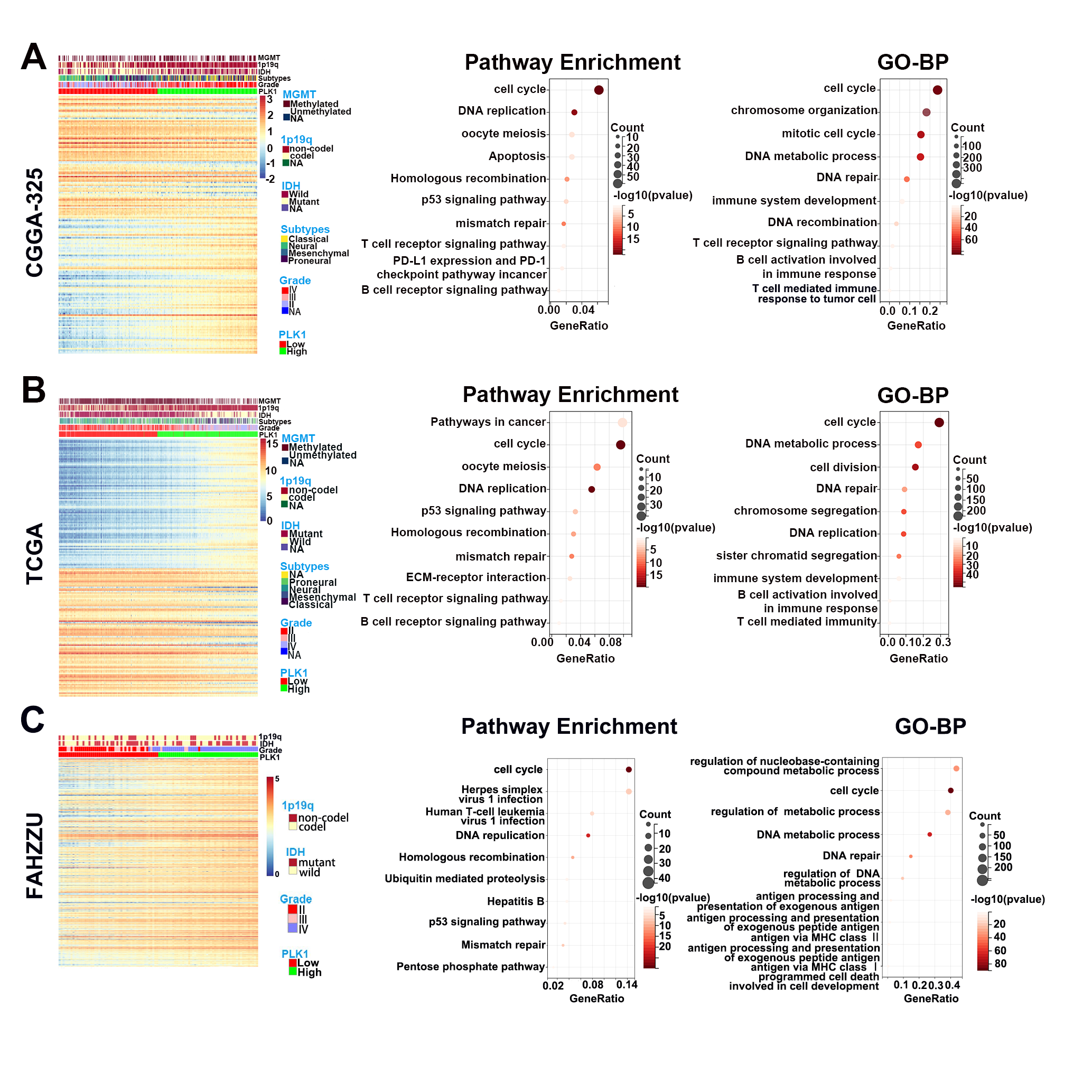

Supplement: Supplementary file 7 [file DataSheet_1.zip › Supplementary Figures/Supplementary Figure 6.tif]

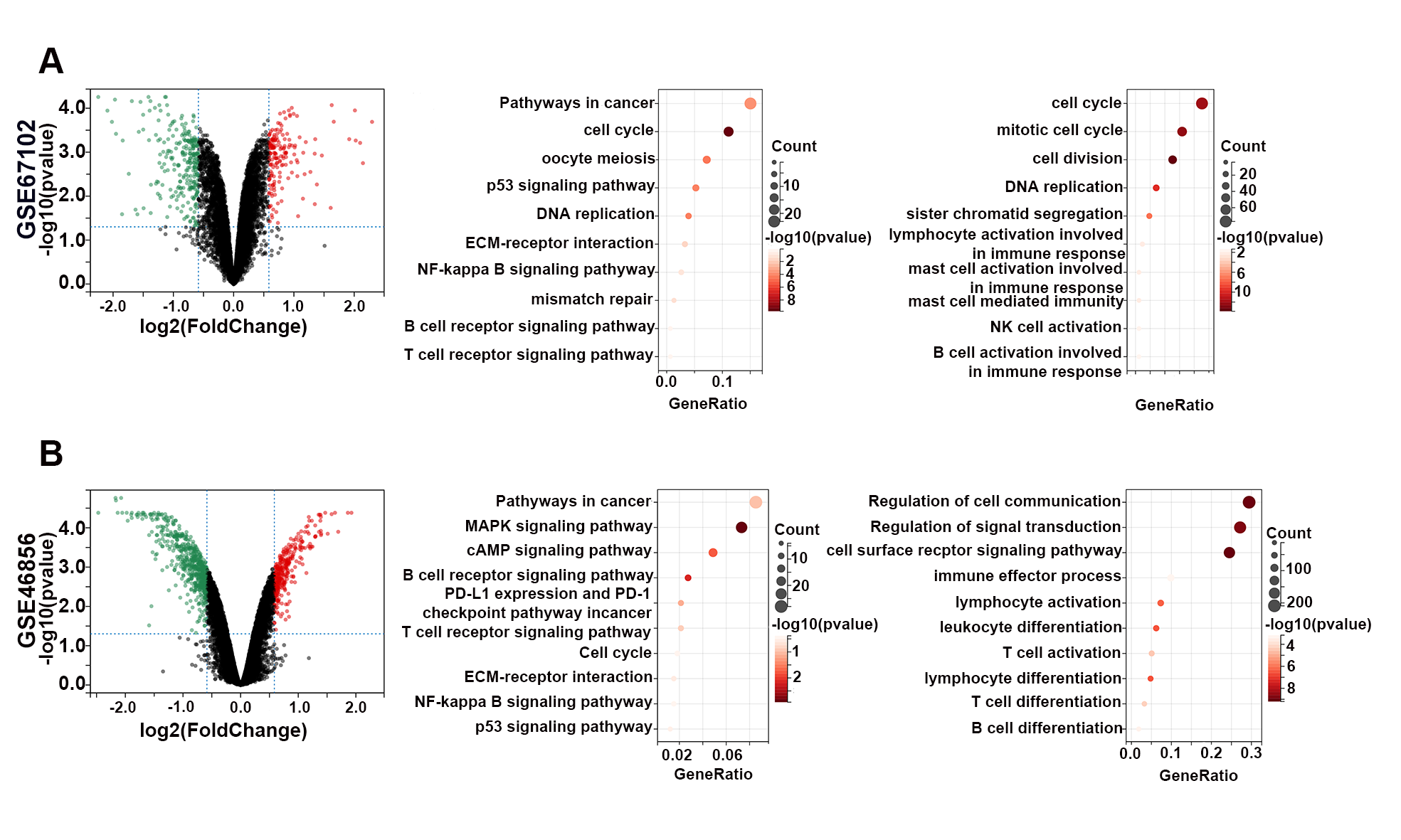

Supplement: Supplementary file 7 [file DataSheet_1.zip › Supplementary Figures/Supplementary Figure 7.tif]

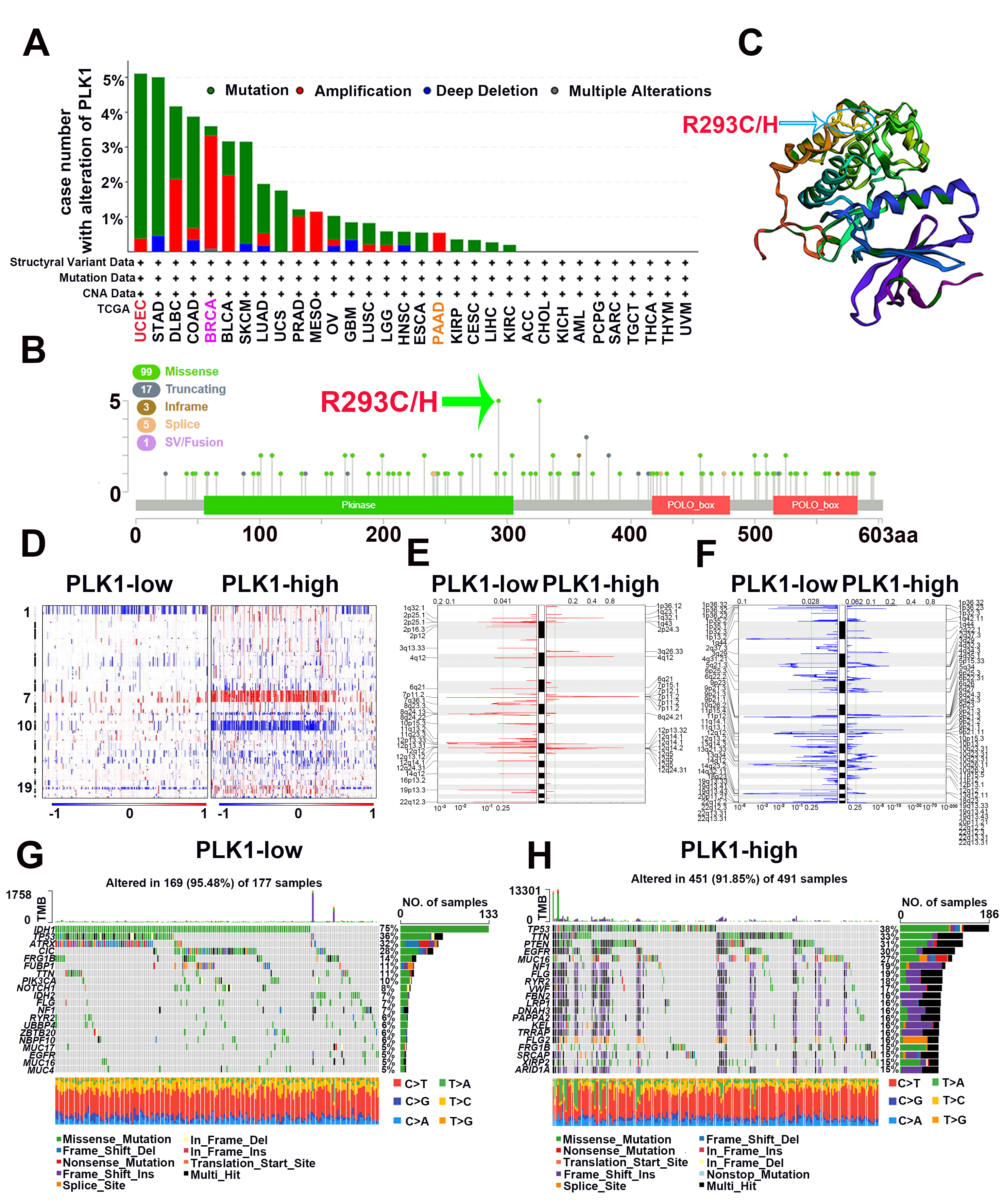

Supplement: Supplementary file 7 [file DataSheet_1.zip › Supplementary Figures/Supplementary Figure 8.tif]

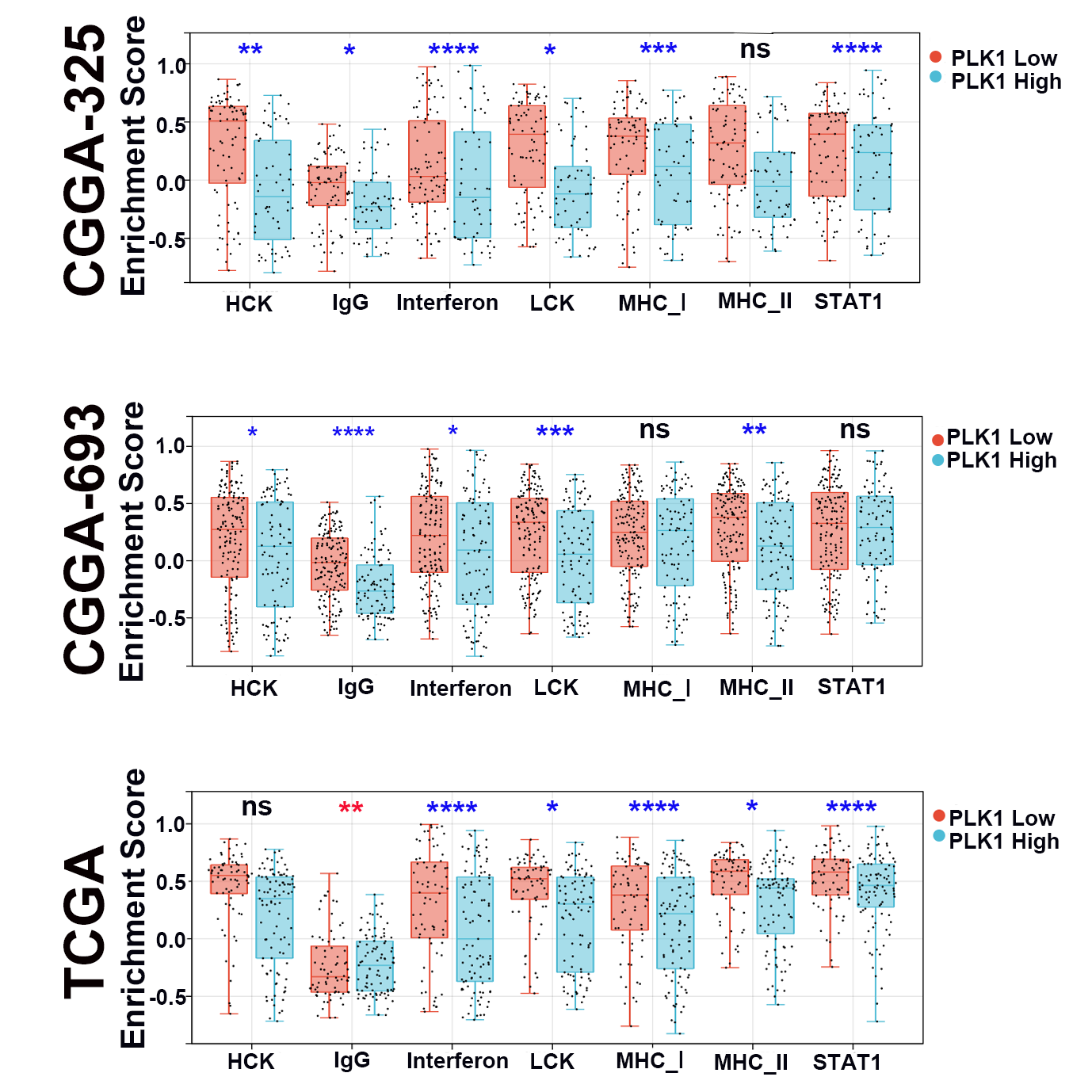

Supplement: Supplementary file 7 [file DataSheet_1.zip › Supplementary Figures/Supplementary Figure 9.tif]
